# Epithelial cell expansion drives cyst progression in genetic models of autosomal recessive polycystic kidney disease

**DOI:** 10.1016/j.isci.2026.116288

**Published:** 2026-06-05

**Authors:** Shuncheng Liu, Xiaole Chen, Zhaoning Liu, Jun Tang, Haoran Tian, Ying Peng, Xiao Mao, Ruping Dai, Bin Zhao, Xugui Li, Li Li, Lingfei Luo, Ming Ma

**Affiliations:** 1 Institute of Developmental Biology and Regenerative Medicine, Southwest University, Beibei, Chongqing 400715, China; 2 School of Fine Arts, Southwest University, Beibei, Chongqing 400715, China; 3 National Health Commission Key Laboratory of Birth Defects for Research and Prevention, Hunan Provincial Maternal and Child Health Care Hospital, Changsha, Hunan 410028, China; 4 Department of Anesthesiology, The Second Xiangya Hospital, Central South University, Changsha, Hunan 410000, China; 5 The Affiliated Hospital of Wuhan Sports University, Wuhan, Hubei 430079, China; 6 Research Center of Stem Cells and Ageing, Chongqing Institute of Green and Intelligent Technology, Chinese Academy of Sciences, Chongqing 400722, China; 7 School of Life Sciences, Fudan University, Shanghai 200433, China; 8 Key Laboratory of Reproductive Health Diseases Research and Translation of Ministry of Education & Key Laboratory of Human Reproductive Medicine and Genetic Research of Hainan Province & Hainan Provincial Clinical Research Center for Thalassemia, The First Affiliated Hospital of Hainan Medical University, Hainan Medical University, Haikou, Hainan 571101, China

**Keywords:** cell biology, transcriptomics

## Abstract

Autosomal recessive polycystic kidney disease (ARPKD) is a pediatric genetic nephropathy caused by mutations in *PKHD1*, which encodes fibrocystin. The cellular basis and epithelial dynamics of cyst formation remain incompletely understood. We used lineage-tracing systems in rat and mouse to define epithelial behavior during cystogenesis. Clonal labeling revealed that renal and biliary epithelial cells undergo marked expansion as cysts form. Genetic mosaic analysis showed that individual *Pkhd1*-deficient cholangiocytes can generate millimeter-scale cysts within one year. Mathematical modeling demonstrated that biliary epithelial proliferation alone can account for cyst growth to millimeter size within weeks to months, depending on initial cell number and doubling time. Transcriptomic profiling of early cystic liver identified upregulated cell-cycle regulators, including CDK1. Pharmacological CDK1 inhibition attenuated fibrocystic liver disease *in vivo*. These findings show that epithelial cell expansion is the primary driver of cystogenesis and loss of fibrocystin activates a pro-proliferative program that can be therapeutically targeted.

## Highlights

• Lineage tracing reveals epithelial expansion drives cyst formation in ARPKD models
• Mosaic analysis shows that an individual *Pkhd1*-null cell is sufficient to form a liver cyst
• Mathematical model shows cell expansion alone can form millimeter-sized liver cyst
• Pharmacological inhibition of CDK1 attenuates fibrocystic liver disease *in vivo*

## Graphical abstract

**Figure:**
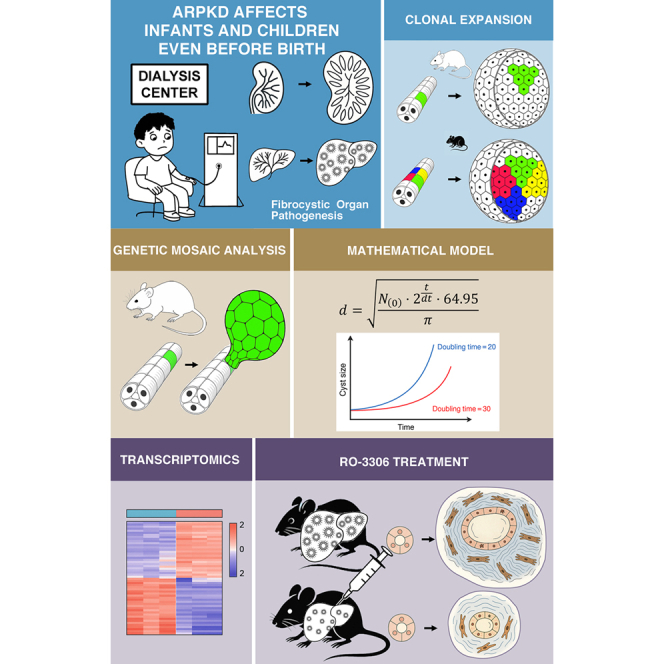

## Introduction

Autosomal recessive polycystic kidney disease (ARPKD) is a rare genetic disorder affecting approximately 1 in 20,000 live births,^1^ characterized by fusiform renal cysts and progressive fibrocystic liver disease. About one-third of affected individuals experience *in utero* or perinatal mortality, and survivors often develop end-stage renal disease in childhood or adolescence. Liver disease can cause portal hypertension and fibrosis, sometimes requiring transplantation.

ARPKD is caused by mutations in *PKHD1*, which encodes fibrocystin (FPC), a large type I transmembrane (TM) protein.^2^ FPC exhibits a complex subcellular localization, including primary cilia, apical membranes, basal bodies, and vesicles overlapping with endoplasmic reticulum and Golgi apparatus markers.^3^^,^^4^^,^^5^^,^^6^ FPC undergoes Notch-like proteolytic processing outside the TM domain, generating a large extracellular domain (ECD), covalently tethered to the TM fragment.^7^ Some models proposed FPC acts as a receptor-like or co-receptor function, while others suggested FPC NTF is released into the lumen^7^ or secreted in exosome-like vesicles that interact with cilia and the cell surface.^8^

FPC has been implicated in the regulation of oriented cell division (OCD), a planar cell polarity (PCP) dependent process that directs longitudinal division along collecting duct morphogenesis.^9^ In rodent models, *Pkhd1* loss randomizes mitotic spindle orientation and may contribute to tubule dilation and cyst formation.^9^^,^^10^^,^^11^ However, *Pkhd1* mutant mice display OCD defects without overt cysts, raising uncertainty about whether OCD disruption alone drives cyst formation.^12^ Several signaling pathways, such as cAMP, IL-8, and YAP, are altered in ARPKD models.^13^^,^^14^^,^^15^ The mechanistic connection between FPC loss and these pathways remains undefined.

Cyst formation in ARPKD involves a significant 3D transformation from renal tubules and bile ducts into cysts, while the epithelial dynamics of cyst formation remain incompletely defined. Recessive loss of *PKHD1* generates cysts, in which all cells have an identical genotype. By tracing the behaviors of individual cells in this process, we can infer the collective behavior of contributing cystogenic cells, and thereby model the process of cyst formation and growth. Lineage tracing enables long-term tracking of cell fate,^16^ while Brainbow/Confetti systems enable multicolor clonal analysis within tissues.^17^ Applying these approaches to ARPKD models provides direct insight into how individual renal and biliary epithelial cells contribute to cyst formation. Several *Pkhd1* mutant mouse models—including null, hypomorphic, and exon-specific deletions—consistently show liver cysts and biliary dysgenesis, but show only minimal renal pathology.^5^^,^^18^^,^^19^ In contrast, the *Pck* rat, an orthologous *Pkhd1* model, displays prominent cysts in both kidney and liver, more closely recapitulating the human ARPKD.^2^ However, the utility of the *PCK* rat for lineage tracing or mosaic analysis to investigate cell behavior during cystogenesis is limited, as this animal model harbors a spontaneous mutation and lacks the necessary genetic tractability.

In this study, we developed new lineage tracing models in rats and mice to dissect cyst initiation and progression in ARPKD. Using tamoxifen-inducible *Pkhd1*^*CreER*^ knock-in rat crossed with *Zs-Green* reporter, and *Brainbow*/*Confetti* labeled *Pkhd1* mutant mice, we tracked the behavior of individual cells during cyst formation in the kidney and liver. We found that the labeled individual cells undergo extensive expansion of cyst-lining epithelia, which is a consistent and progressive feature of disease. To examine the cystogenic capacity of individual homozygous *Pkhd1* mutant cells, we generated *Pkhd1* homozygous cells in the background of heterozygous cells and found that the individual homozygous cells undergo expansion and form millimeter-scale cysts within a year. Based on this, a mathematical model indicated that cell proliferation alone is sufficient to develop into millimeter-sized cysts within weeks or months, depending on the initial cell number of participating cells and doubling time (***dt***). Transcriptomic profiling of early-stage cystic liver tissue identified *Cdk1* as a key proliferative driver, and pharmacological inhibition of Cdk1 with RO-3306 significantly reduced liver cyst growth and fibrosis. These findings provide direct *in vivo* evidence that epithelial cell expansion drives cyst formation in ARPKD and suggest that targeting downstream pro-proliferative signaling could be a therapeutic strategy for ARPKD.

## Results

### Generation and characterization of *Pkhd1*^*CreER*^ rats: Recombinase activity in renal tubular epithelial cells and cholangiocytes

**Figure 1:**
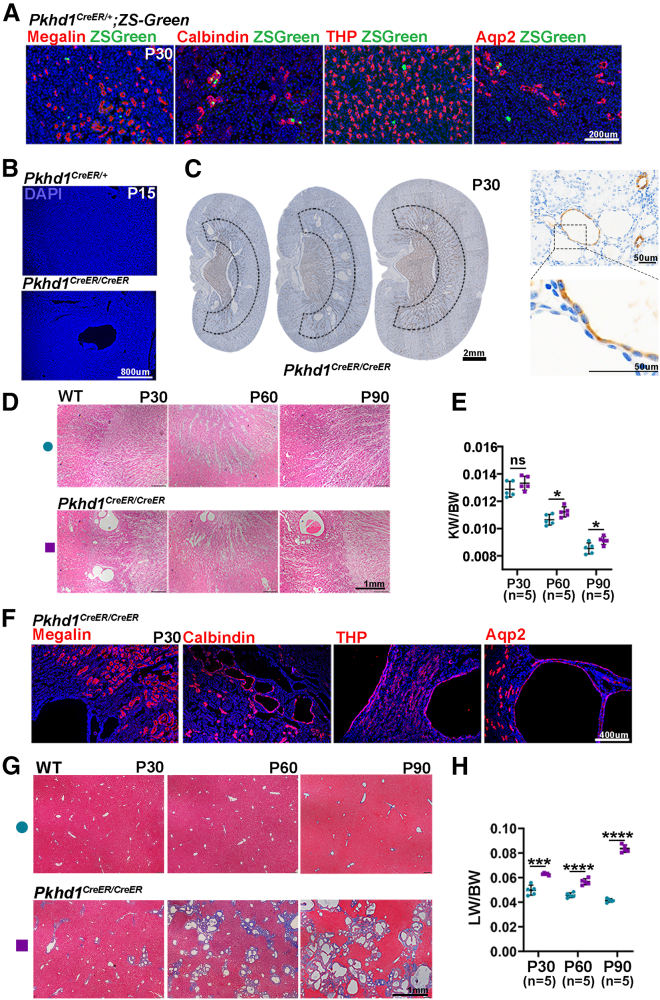
Development and characterization an inducible Cre recombinase *Pkhd1*^*CerER*^ knock-in rat, and cyst formation in *Pkhd1*^*CreER/CreER*^ rats (A) Immuno-fluorescence with anti-Megalin, anti-Calbindin, anti-Tamm Horsfall protein, and anti-Aqp2 antibodies on *Pkhd1*^*CreER/+*^;*ZS-Green* rat kidney sections at P30, which were induced with tamoxifen at P5. (B) *Pkhd1*^*CreER/+*^ and *Pkhd1*^*CreER/CreER*^ kidney sections were stained with DAPI to reveal that kidneys develop cysts before P15. (C) Scanned image of kidney sections from 3 *Pkhd1*^*CreER/CreER*^ rats, immuno-histochemistry with anti-Aqp2 antibody at P30, cyst development in the cortico-medullary region. Right: Enlargement of the cystic area. (D) H&E-stained kidney sections of WT and *Pkhd1*^*CreER/CreER*^ rats at P30, P60, and P90. (E) Aggregated data of kidney body weight ratio of WT (*n* = 5 at each time point) and *Pkhd1*^*CreER/CreER*^ (*n* = 5 at each time point) rats at P30, P60, and P90. (F) Immuno-fluorescence with anti-Megalin, anti-Calbindin, anti-Tamm Horsfall protein antibody, and anti-Aquaporin 2 antibody on the kidney sections of *Pkhd1*^*CreER/CreER*^ rats at P30. (G and H) Masson-Trichrome staining of rat liver sections (G) and aggregated data of liver body weight ratio (H) of WT (*n* = 5 at each time point) and *Pkhd1*^*CreER/CreER*^ (*n* = 5 at each time point) at P30, P60, P90. Error bars represent mean ± s.e.m, ∗*p* < 0.05, ∗∗*p* < 0.01, and ∗∗∗*p* < 0.001 by two-way ANOVA (E and H). Scale bars, 200 μm (A), 800 μm (B), 2 mm (C), 50 μm (C right), 400 μm (F), and 1 mm (D and G).

To enable lineage tracing in ARPKD, we first developed a tamoxifen-inducible Cre knock-in rat model, *Pkhd1*^*CreER*^, in which exons 2–5 of *Pkhd1* were replaced with a *CreERT2-polyA* cassette (Figure S1A). This design disrupts *Pkhd1* function while placing *CreER* under the control of endogenous *Pkhd1* regulatory elements. Correct targeting was confirmed by PCR using tail genomic DNA followed by Sanger sequencing (Figures S1A and S1B). RT-PCR analysis using kidney cDNA verified *CreERT2* mRNA expression in both *Pkhd1*^*CreER/+*^ and *Pkhd1*^*CreER/CreER*^ rats (Figure S1B). The responsiveness of *Zs-Green* reporter rats^20^ was verified by breeding with *CAG-NCre* rats,^21^ which drives constitutive Cre recombinase expression. Extensive ZsGreen signals were observed throughout the kidney and liver sections of *CAG-NCre*;*Zs-Green rats*, demonstrating the effectiveness of this reporter system for lineage tracing (Figures S1C and S1D). To examine *Pkhd1*^*CreER*^-driven Cre activity, we crossed *Pkhd1*^*CreER*^ rats with *Zs-Green* reporter rats. In *Pkhd1*^*CreER/+*^;*ZsGreen* rats, tamoxifen administration at postnatal day 5 (P5) induced sporadic ZsGreen expression that was localized to renal collecting ducts and outside the collecting duct, whereas it was exclusively restricted to cholangiocytes in the liver, reflecting the low intrinsic efficiency of the *Pkhd1* promoter. Conversely, vehicle-injected controls showed no signal, confirming tight regulation of the system and the absence of leaky expression (Figures S1E and S1F). Attempts to enhance recombination by daily tamoxifen injections on P5 and P6 were poorly tolerated, with no survival after three consecutive injections at P5-P7, and administration at P3 or earlier resulted in high mortality, which was primarily due to the low tolerance of rat pups to the intraperitoneal injection of corn oil. We isolated primary kidney cells from *Pkhd1*^*cre/+*^;*ZsGreen* rats at P3 and treated them with varying concentrations of 4-OH tamoxifen, 1–5 μM induce ZsGreen expression, and 10 μM treatment or above reduced cell survival (Figure S2). Co-staining with Aqp2 revealed ZsGreen expression cells in the collecting ducts as well as additional nephron segments (Figure 1A). Co-staining of segment-specific markers at P30 further confirmed ZsGreen-positive cells in proximal tubules (Megalin), thick ascending limbs (THP), and distal convoluted tubules (calbindin), indicating broad nephron targeting by *Pkhd1*^*CreER*^-driven recombination (Figure 1A).

### *Pkhd1*^*CreER/CreER*^ rats develop cysts in the kidney and liver

We examined cyst onset and progression in *Pkhd1*^*CreER/CreER*^ rats. No renal cysts were observed at P7. By P15, microcysts were detected in two out of three rats (Figure 1B). At P30, all three examined rats exhibited both Aqp2^+^ and Aqp2^-^ cysts, with one animal showing more advanced disease than the others. Despite the uniform Sprague-Dawley (SD) genetic background, this indicates variable onset and progression in *Pkhd1*^*CreER/CreER*^ rat (Figure 1C). From P30 to P90, H&E staining revealed slow cyst expansion (Figures 1D, 1E, and S4), and even at 1 year, only a few large cysts were observed near the cortico-medullary junction (Figures S3A and S3B). Segmental marker analysis showed that cyst-lining cells expressed Aqp2, THP, and Calbindin, but not Megalin, indicating cyst origin from the collecting duct, thick ascending limb, and distal convoluted tubule, but not proximal tubule (Figure 1F). Thus, renal cysts arise around P15, progress slowly, and display highly inter-animal variability, with few tubules affected.

In contrast, hepatic cysts were evident at birth. Between P30 and P90, cysts enlarged progressively and were accompanied by fibrotic changes (Figures 1G, 1H, and S4). Nearly all bile ducts became cysts, in stark contrast to the sparse renal phenotype. At 1 year, the rats develop severe polycystic liver disease (Figures S3C and S3D). These results demonstrate that *Pkhd1*^*CreER/CreER*^ rats recapitulate fibrocystic liver disease more robustly than kidney disease, consistent with observations in *PCK* rat.^10^

### Lineage tracing reveals individual renal tubule cells undergo expansion in cystogenesis in *Pkhd1*^*CreER/CreER*^;*ZsGreen* rats

**Figure 2:**
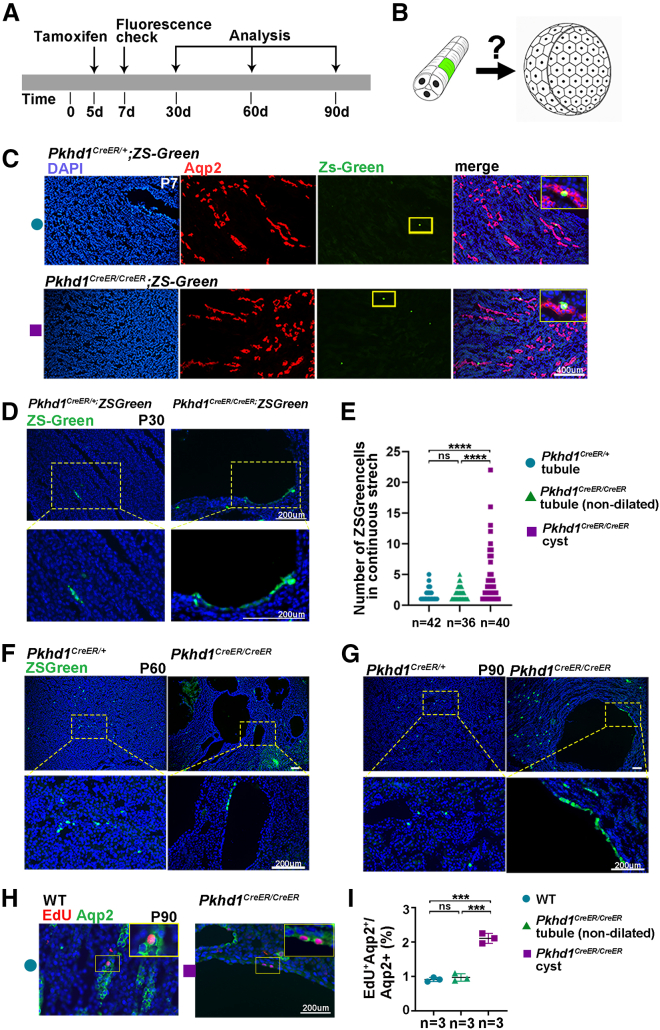
Lineage tracing renal epithelial cells in cyst growth in *Pkhd1*^*CreER/CreER*;^*ZS-Green* rats (A and B) Schematic drawing of the design of lineage tracing, individual cells were labeled at the tubule stage before cyst formation. (C) Immuno-fluorescence of anti-Aqp2 (red) on kidney sections of *Pkhd1*^*CreERl+*^;*ZS-Green* and *Pkhd1*^*CreERlCreER*^;*ZS-Green* rats at P7 induced with corn oil or tamoxifen with a dose of 200 mg/kg intraperitoneally injected at P5. (D) Images of kidney sections of *Pkhd1*^*CreER/+*^;*ZS-Green* (*n* = 3) and *Pkhd1*^*CreER/CreER*^;*ZS-Green* (*n* = 7) rats at P30 induced with tamoxifen at P5. (E) Aggregated data on the number of ZSGreen cells in continuous stretch in the tubules of *Pkhd1*^*CreER/+*^;*ZS-Green* (*n* = 42), non-dilated tubules of *Pkhd1*^*CreER/CreER*^;*ZS-Green* (*n* = 36), and cysts of *Pkhd1*^*CreER/CreER*^;*ZS-Green* (*n* = 40) kidney. Continuous stretch from 3 *Pkhd1*^*CreER/+*^;*ZS-Green* and 7 *Pkhd1*^*CreER/CreER*^;*ZS-Green* rats. (F and G) Images of kidney sections of *Pkhd1*^*CreER/+*^;*ZS-Green* (*n* = 3) and *Pkhd1*^*CreER/CreER*^;*ZS-Green* (*n* = 3) rats at P60 (F) and P90 (G) induced with tamoxifen at P5. (H) Immuno-fluorescence using anti-EdU (red) and anti-Aqp2 (green) antibodies on the sections of WT and *Pkhd1*^*CreER/CreER*^ rats injected with EdU 3 h before sacrifice. (I) Aggregated data of the ratio of EdU+ nuclei in the control and cyst lining Aqp2+ cells in WT (*n* = 3) and *Pkhd1*^*CreER/CreER*^ (*n* = 3) rats at P90. Error bars represent mean ± s.e.m, ∗*p* < 0.05, ∗∗*p* < 0.01, and ∗∗∗*p* < 0.001 by two-way ANOVA (E and I). Scale bars, 400 μm (C); 200 μm (D, F, G, and H).

To examine epithelial cell behavior during cyst formation, we performed lineage tracing using *Pkhd1*^*CreER/CreER*^;*ZsGreen* rats (Figures 2A and 2B). A single dose of tamoxifen was administered at postnatal day 5 (P5) to *Pkhd1*^*CreER/CreER*^;*ZsGreen and Pkhd1*^*CreER/+*^;*ZsGreen control rats*, and individual scattered renal tubule cells were labeled at P7 prior to cyst initiation. As expected, two copies of CreER in *Pkhd1*^*CreER/CreER*^ *rats* generated more ZSGreen cells than *Pkhd1*^*CreER/+*^ *rats;* however, labeled ZSGreen cells remain isolated and scattered at this stage (Figure 2C). At P30, *Pkhd1*^*CreER/CreER*^;*ZsGreen* rats developed a small number of renal cysts. Although the low recombination efficiency of *Pkhd1*^*CreER*^ resulted in sparse initial labeling, ZSGreen-positive cells formed large, contiguous stretches along the cyst-lining epithelium in cystic regions. In contrast, labeled clones in neighboring non-cystic tubules and in *Pkhd1*^*CreER/+*^; *ZsGreen* controls typically contained only one to two cells. (Figures 2D and 2E). This pattern persisted at P60 and P90, with extensive ZSGreen cell stretches lining cysts (Figures 2F and 2G). We define *Pkhd1*-deficient cells destined to develop into cysts as cystogenic cells. Our data indicate that these cells undergo expansion during the transition from tubules to cysts. These results suggest that the proliferation rate is higher in cystic tubules compared to non-cystic tubules or *Pkhd1*^*CreER/+*^ cells, but *Pkhd1* deletion does not necessarily lead to higher proliferation before cyst formation. Certainly, more than one cell contributes to each cyst, and we could infer that a group of neighboring cystogenic tubular cells proliferate and expand laterally and increase the diameter of the lumen of cysts, which is the epithelial dynamics of cystogenesis. To quantify proliferation, we administered 5-ethynyl-2′-deoxyuridine (EdU) to P90 rats 3 h prior to sacrifice and performed co-staining with anti-Aqp2 antibody. The proportion of EdU+ cells among Aqp2+ cyst-lining epithelia was ∼2% in cysts, compared to ∼1% in non-cystic tubules or controls. (Figures 2H and 2I), supporting increased proliferation in cystic cells.

### Lineage tracing of collecting duct cell-oriented cell division in *Pkhd1*^*CreER/CreER*^;*ZsGreen* rats and evaluate their contribution to cyst formation

**Figure 3:**
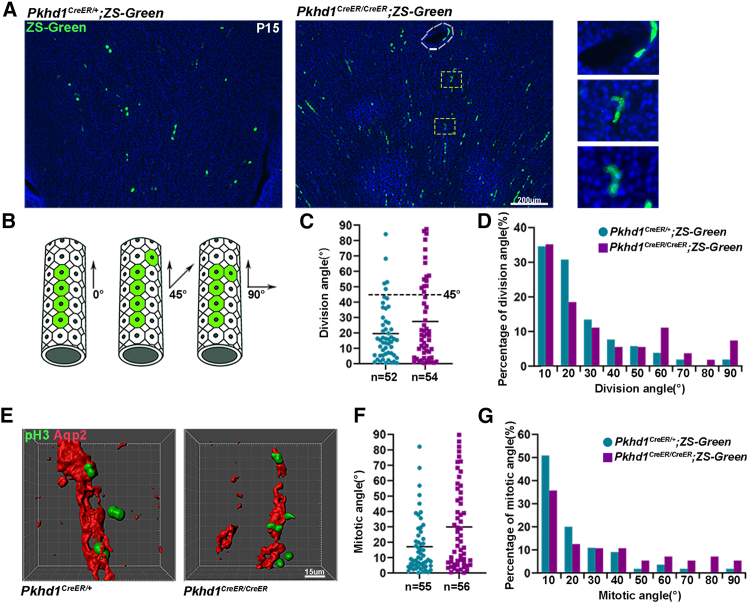
Examination of oriented cell division for tubulogenesis of the collecting duct and cyst formation in *Pkhd1*^*CreER/CreER*^;*Zs-Green* rats via lineage tracing (A) Images of the medulla on kidney sections of *Pkhd1*^*CreER/+*^;*ZS-Green* (*n* = 3) and *Pkhd1*^*CreER/CreER*^;*ZS-Green* (*n* = 3) rats at P15 induced with tamoxifen at P5. Dashed white line circles a microcyst in the cortico-medullary region. Dashed yellow square boxes illustrate the transverse division of collecting duct cells. (B) Schematic drawing of the division angle of 0°, 45° and 90 degree of the collecting duct epithelial cells via lineage tracing. (C and D) Aggregated data on division angle of tubules from *Pkhd1*^*CreER/+*^;*ZS-Green* (*n* = 52 from 7 rats), and tubules from *Pkhd1*^*CreER/CreER*^;*ZS-Green* (*n* = 54 from 5 rats) (*p* = 0.2665, Mann-Whitney *U* test). (E) 3D reconstruction of the renal collecting ducts in the kidneys of *Pkhd1*^*CreER/+*^ (left) and *Pkhd1*^*CreER/CreER*^ (right) rats. The tubule-axis coordinates were represented by immunofluorescence staining with anti-Aqp2. Separating chromosomes was represented by anti-H3pS10 labeling. (F and G) Aggregated data of mitotic angles in *Pkhd1*^*CreER/+*^ rats (*n* = 55 from 5 rats) and *Pkhd1*^*CreER/CreER*^ rats (*n* = 56 from 5 rats) (*p* = 0.0168, Mann-Whitney *U* test). Rats were sacrificed between P10 and P15. Scale bars, 200 μm (A); 15 μm (E).

Our lineage tracing approach also enabled us to re-evaluate the contributions of OCD for cyst formation. We administered tamoxifen at P5 to *Pkhd1*^*CreER/+*^;*Zs-Green* and *Pkhd1*^*CreER/CreER*^;*Zs-Green* rats, and examined kidneys at P15. At this stage, microcysts have developed in the cortico-medullary region (Figure 3A). To assess OCD, we examined the collecting duct in the medulla via coronal and sagittal sections. In both *Pkhd1*^*CreER/+*^;*Zs-Green* and *Pkhd1*^*CreER/CreER*^;*Zs-Green* rats, we measured and categorized the division angles of labeled cell clones (Figure 3B). We found a tendency for a higher proportion of transverse divisions in *Pkhd1*^*CreER/CreER*^;*Zs-Green* rats than that of heterozygous controls (Figures 3C and 3D). Furthermore, 3D reconstruction of confocal images of coronal sections co-stained with anti-phospho-Histone 3 (p-H3) and anti-Aqp2 antibodies revealed that the angle of the mitotic spindle was more significantly transverse in *Pkhd1*^*CreER/CreER*^ kidneys than in heterozygous rats (Figures 3E–3G). Collectively, these findings indicate that a disruption in PCP—a key regulator of OCD—in a subset of collecting ducts upon *Pkhd1* impairment. However, this loss of OCD alone appears insufficient to drive cyst formation.

### Lineage tracing of biliary cells in *Pkhd1*^*CreER/CreER*^;*ZsGreen* rats

**Figure 4:**
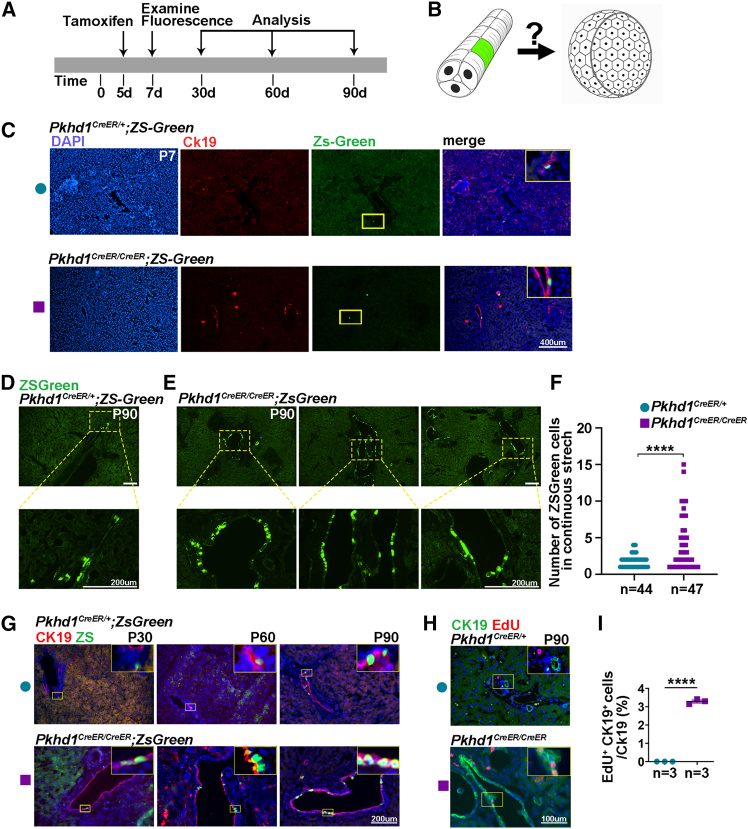
Lineage tracing cholangiocytes in cyst growth in *Pkhd1*^*CreER/CreER*;^*ZS-Green* rats (A and B) Schematic drawing of the experimental design, individual cells were labeled with ZSGreen at the dilated tubule or early cystic stage and examined the signal at later advanced cystic stages. (C) Immuno-fluorescence of anti-CK19 (red) on liver sections of *Pkhd1*^*CreERl+*^;*ZS-Green* and *Pkhd1*^*CreERlCreER*^;*ZS-Green* rats at P7 induced with tamoxifen intraperitoneally injected with a dose of 200 mg/kg into experiment rats at P5. (D and E) Images of liver sections of *Pkhd1*^*CreER/+*^;*ZS-Green* (D) (*n* = 3) and *Pkhd1*^*CreER/CreER*^;*ZS-Green* (E) (*n* = 3) rats at P90 induced with tamoxifen at P5. (F) Aggregated data on the number of ZSGreen cells in continuous stretch in the bile ducts of *Pkhd1*^*CreER/+*^;*ZS-Green* (*n* = 44) and cysts of *Pkhd1*^*CreER/CreER*^;*ZS-Green* (*n* = 47) liver. Continuous stretch from 3 *Pkhd1*^*CreER/+*^;*ZS-Green* and 3 *Pkhd1*^*CreER/CreER*^;*ZS-Green* rats. (G) Immuno-fluorescence with anti-CK19 (red) antibody on liver sections of *Pkhd1*^*CreER/+*^;*ZS-Green* and *Pkhd1*^*CreER/CreER*^;*ZS-Green* rats at P30, P60, and P90 induced with tamoxifen at P5. (H and I) Immuno-fluorescence using anti-EdU and anti-CK19 antibodies (H) and aggregated data of the ratio of EdU positive cells in the bile duct cells (I) on the section of *Pkhd1*^*CreER/+*^ and *Pkhd1*^*CreER/CreER*^ rats injected with EdU 3 h before sacrifice. Error bars represent mean ± s.e.m, ∗*p* < 0.05, ∗∗*p* < 0.01, and ∗∗∗*p* < 0.001 by Student’s *t* test (F and I). Scale bars, 400 μm (C); 200 μm (D, E, and G); 100 μm (H).

To study cholangiocyte behaviors during ARPKD cyst progression, we performed lineage tracing in *Pkhd1*^*CreER/CreER*^;*ZsGreen* rats. Masson’s trichrome staining revealed progressive cyst enlargement and fibrosis between P30 and P90 (Figures 1G and 1H). These rats were intraperitoneally injected with a single dose of tamoxifen at P5. We found that individual scattered cells were labeled on CK19-positive cells at P7 (Figures 4A–4C), and we analyzed fluorescence at P30, P60, and P90 (Figures 4D–4G). In *Pkhd1*^*CreER/CreER*^;*ZsGreen* rats, ZsGreen cells formed continuous stretches along the cyst-lining epithelium, whereas in *Pkhd1*^*CreER/+*^;*ZsGreen* controls, clones typically contain only one to two cells (Figures 4D–4F), indicating clonal expansion of cyst-lining epithelial cells. To assess proliferation, we administered EdU to P90 rats 3 h prior to sacrifice and performed co-staining with anti-CK19. In cystic epithelia marked by CK19, the percentage of EdU+ nuclei increased to ∼3% in *Pkhd1*^*CreER/CreER*^ rats, compared to ∼0% in controls (Figures 4H and 4I), supporting enhanced proliferation in *Pkhd1* mutant cholangiocytes. Together, these results demonstrate that individual cholangiocytes undergo expansion, and the proliferation rate is higher, in liver cyst formation in ARPKD.

### Multicolor analysis of cholangiocytes for cyst formation in the liver of *Pkhd1*^−/−^ mice

**Figure 5:**
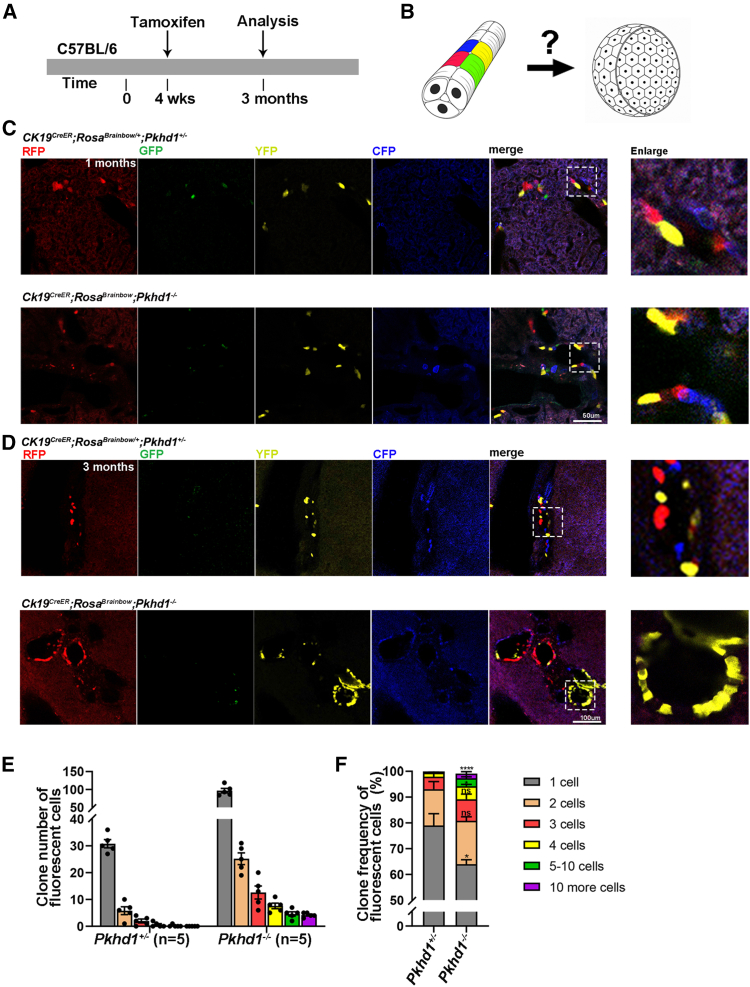
Lineage tracing cholangiocytes in cyst growth in a mouse model of ARPKD using *Rosa*^*Brainbow*^ mice (A and B) Schematic drawing of the experimental design, individual cells were labeled with distinct fluorophores at the early cystic stage, and the fluorescence was examined at a more advanced cystic stage. (C) Confocal images with GFP, YFP, BFP, and RFP channels of the liver section of *CK19*^*CreER*^;*Pkhd1*^*+/−*^;*Rosa*^*Brainbow/+*^ and *CK19*^*CreER*^;*Pkhd1*^−/−^;*Rosa*^*Brainbow/+*^ mice at P30 with a single dose of tamoxifen at P28. (D) Confocal images with GFP, YFP, BFP, and RFP channels of the liver section of *CK19*^*CreER*^;*Pkhd1*^*+/−*^;*Rosa*^*Brainbow/+*^ and *CK19*^*CreER*^;*Pkhd1*^−/−^;*Rosa*^*Brainbow/+*^ mice at P90 with a single dose of tamoxifen at P28. (E and F) Clonal number (E) and clonal frequency (F) of each fluorophore in *CK19*^*CreER*^;*Pkhd1*^*+/−*^;*Rosa*^*Brainbow/+*^ and *CK19*^*CreER*^;*Pkhd1*^−/−^;*Rosa*^*Brainbow/+*^ mice at P90, data were collected from 5 mice of each genotype. Error bars represent mean ± s.e.m, ∗*p* < 0.05, ∗∗*p* < 0.01, and ∗∗∗*p* < 0.001 by two-way ANOVA (F). Scale bars, 50 μm (C); 100 μm (D).

Because bile ducts remodel postnatally, lineage tracing at P5 reflects both developmental and cystic growth. To focus on cyst dynamics, we performed lineage tracing in adult mice during the cyst growth phase. We employed the *Rosa*^*Brainbow2*.*1*^ multicolor reporter system, which stochastically labels cells with distinct fluorophores upon Cre-mediated recombination (Figure S5A). Using a CRISPR/Cas9-generated *Pkhd1*^−/−^ mouse model with the deletion of exons 4 and 5, genotyping confirmed the targeted deletion (Figures S5B–S5D). As expected, *Pkhd1*^−/−^ mice developed progressive polycystic liver disease but did not exhibit cystic kidney pathology (Figures S5E and S5F). To perform cholangiocytes specific lineage tracing in *Pkhd1*-deficient background, we crossed *Ck19*^*CreER*^ mice with *Pkhd1*^−/−^ and *Rosa*^*Brainbow*^ reporter mice and generated *Ck19*^*CreER*^;*Pkhd1*^−/−^;*Rosa*^*Brainbow/+*^ and control *Ck19*^*CreER*^;*Pkhd1*^*+/−*^;*Rosa*^*Brainbow/+*^ mice. A single tamoxifen dose was administered at P28, and livers were collected at 3 months (Figures 5A and 5B). We prepared 50-μm vibratome sections and performed serial confocal imaging to capture all four *Brainbow*-encoded fluorophores. First, we examined the efficiency of *CK19*^*CreER*^ three days after tamoxifen administration. The results showed that it could produce four discontinuous fluorescent signals in the cholangiocytes of *Pkhd1*^*−/+*^ and *Pkhd1*^−/−^ mice (Figure 5C). z stack 3D reconstructions revealed that, although not all cyst-lining cells were labeled, discrete epithelial patches expressing the same fluorophore were present (Figure 5D; Videos S1 and S2). We noted an imbalance in the proportion of fluorescent labels, specifically a skewed distribution of CFP and GFP signals (Figures 5C and 5D). This phenomenon is a known limitation of the *Rosa*^*Brainbow2*.*1*^ system, attributed to differences in fluorophore brightness and stochastic biases in Cre-mediated recombination.^22^ Each patch of cells most likely represents the cells derived from single epithelial cell among other cells of functionally identical genotype, we counted the number cells within each distinct color patches and calculated the frequency of each fluorophore in *CK19*^*CreER*^;*Pkhd1*^*+/−*^;*Rosa*^*Brainbow/+*^ and *CK19*^*CreER*^;*Pkhd1*^−/−^;*Rosa*^*Brainbow/+*^ mice, and found that the frequency of continuous fluorescent cell clusters appearing was significantly higher in the biliary cysts of mutant mice suggests loss of *Pkhd1* leads to cholangiocytes expansion (Figures 5E and 5F). Proliferation rates in bile duct cyst-lining cells were significantly higher than those in wild-type controls (Figures S5G and S5H). These data indicate that the labeled cholangiocytes undergo expansion in polycystic liver disease.

Video S1. 3D reconstruction with Z-stack in Pkhd1+/−;Rosabrainbow/+;CK19CreER mouse liver at P90, related to Figure 5

Video S2. 3D reconstruction with Z-stack in Pkhd1−/−;Rosabrainbow/+;CK19CreER mouse liver at P90, related to Figure 5

### Generation of *Pkhd1*^*fl*^ conditional rat and evaluate cystogenic potential of individual *Pkhd1*^*CreER/-*^ homozygous cholangiocyte

Lineage tracing demonstrated that individual renal epithelial cells and cholangiocytes undergo expansion in the process of cyst progression; each cyst is derived from multiple cells. To examine the cystogenic potential of individual homozygous cells, we generated *Pkhd1* homozygous mutant cells among heterozygous cells, and examined whether individual cells are sufficient to generate cystic structures.

We generated a conditional *Pkhd1*^*fl*^ allele with *LoxP* sites flanking exons 14–16, such that Cre excision produced a null allele (Figure S6A). Using CRISPR/Cas9-mediated targeting in zygotes, we inserted the *LoxP* sites and confirmed correct targeting by PCR and Sanger sequencing (Figure S6B). We then validated Cre responsiveness by crossing *Pkhd1*^*fl*^ rats to *CAG*-*NCre* transgenic rats. PCR analysis of kidney cDNA showed that Cre recombination ablated exons 14–16, confirming the generation of *Pkhd1*^*-*^ alleles (Figure S6C). We generated *Pkhd1*^−/−^ rats and characterized the polycystic kidney disease phenotype at P45, P90, P270, and P360. *Pkhd1*^−/−^ rats develop macroscopic cysts that are obvious in the medulla and cortical-medullary region at P45 and P90, a few macroscopic cysts are present at the cortical-medullary region at P270 and P360 (Figure S6D). At all time points analyzed, both the kidney body weight ratio (KW/BW) and the cystic index (CI) were significantly elevated in *Pkhd1*^−/−^ rats compared to WT controls (Figure S6E). Despite the structural abnormalities, serum urea nitrogen, and creatinine levels in 1-year-old *Pkhd1*^−/−^ rats were indistinguishable from those of control littermates, indicating preserved renal function (Figure S6E). Notably, the KBW ratios were comparable between males and females, indicating that the renal phenotype is independent of gender (Figure S6F). HE staining of kidney sections shows the cysts are from the cortical-medulla region (Figures S6D and S6G). To characterize which segments generate cysts in *Pkhd1* mutant rats, we used anti-Megalin, anti-Aqp2, and anti-Tamm-Horsfall protein (THP) antibodies to mark the proximal tubule, the collecting duct, and the thick ascending limb of Henle’s loop (TALH), respectively. Cysts stained positive for Aqp2 and THP but negative for Megalin, indicating that *Pkhd1*^−/−^ mutant cysts are from the collecting duct and the thick ascending limb of Henle’s loop (Figure S6H). We further examined the cellular changes within the cysts. EdU assay performed at the advanced cystic stage (P360) revealed a minimal proliferation rate in cyst lining cells (Figures S6I and S6J). We used the TUNEL assay to examine the rate of apoptosis; however, we did not find elevated apoptosis in the collecting duct (Figure S6K). For cell shape change, we used anti-Laminin to mark the basement membrane of the tubule/cysts, and anti-Aqp2 to mark the apical and lateral sides of the cyst lining cells. Double staining clearly revealed that cyst lining cells are flattened in the cystic stage from a columnar shape in non-cystic control kidneys (Figure S6L).

We also characterized the hepatic phenotype of the mutant rats at multiple periods. HE staining and liver body weight ratio demonstrated a progressive exacerbation of the polycystic liver phenotype from P45 to P365 (Figures S7A and S7B). The hepatic phenotype is independent of gender (Figure S7C). For fibrosis, Trichrome-Masson staining of the mutant’s liver sections is indistinguishable from the WT control at P0, and collagen deposition around the cysts is prominent in the mutants at P45 and P180 (Figure S7D). We further examined the expression of fibrotic markers (*Tgfb1*, *Col1a1*, *Col3a1*, *Fn1*, *Acta2*, and *Timp1*) by RT-qPCR at P45. Among these, *Col1a1*, *Col3a1*, *Acta2*, and *Timp1* expression were significantly upregulated in *Pkhd1*^−/−^ (Figure S7E). We next examined smooth muscle actin (SMA) and collagen type III alpha 1 chain (Col3a1) expression by immunofluorescence with anti-SMA and anti-Col3a1 antibodies. SMA expression is moderately increased in the mutants, and strong Cola3a1 expression surrounds cysts in the mutants (Figure S7G). Western blot of whole liver protein extraction also confirmed that SMA protein expression is increased in *Pkhd1*^−/−^ at P45 (Figure S7F). In conclusion, *Pkhd1*^−/−^ rats developed renal and hepatic cysts with preserved kidney function and progressive fibrocystic liver disease.

**Figure 6:**
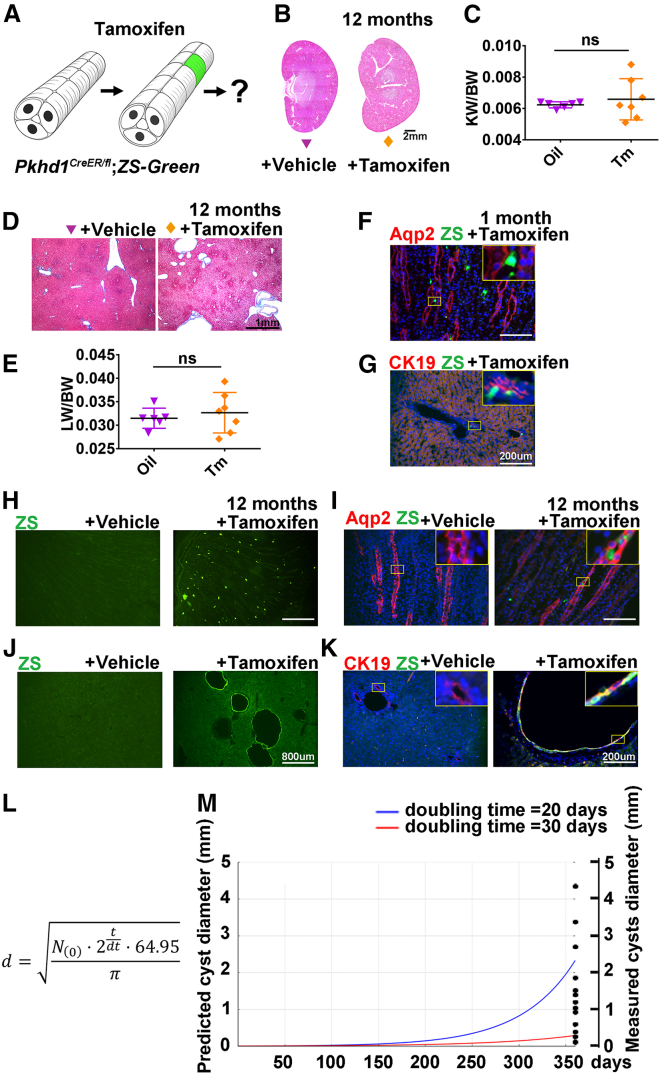
Examine cystogenic potential of individual *Pkhd1* homozygous cells in the background of heterozygous cells in *Pkhd1*^*CreER/fl*^ rat, and model cyst growth dynamics (A) Schematic drawing of the experimental design of analysis, the green labeled cell represents *Pkhd1*^*CreER/-*^;*ZsGreen* cell to assay the cystogenic potential of individual *Pkhd1* homozygous cells. (B and C) Hematoxylin and eosin staining of the 1-year-old rat kidney sections (B) and aggregated kidney body weight ratio (C) of *Pkhd1*^*CreER/fl*^ rat induced with corn oil (*n* = 6) or tamoxifen (*n* = 7). (D and E) Masson-Trichrome staining of 1-year-old rat liver sections (D) and the liver body weight ratio (E) of *Pkhd1*^*CreER/fl*^;*ZS-Green* induced with corn oil (*n* = 6) or tamoxifen (*n* = 7). (F) Immuno-fluorescence with anti-Aqp2 (red) antibody on the kidney section of *Pkhd1*^*CreER/fl*^;*ZS-Green* rats at 1-month-old induced with tamoxifen at P5 and P6. (G) Immuno-fluorescence with anti-Ck19 (red) antibody on liver sections of *Pkhd1*^*CreER/fl*^;*ZS-Green* rats at 1-month-old induced with tamoxifen at P5 and P6. (H) Kidney section of a 12-month-old *Pkhd1*^*CreER/fl*^;*ZS-Green* rat induced with corn oil or tamoxifen at P5 and P6. (I) Immuno-fluorescence with anti-Aqp2 (red) antibody on kidney sections of 12-month-old *Pkhd1*^*CreER/fl*^;*ZS-Green* rat and counterstained with DAPI, induced with corn oil or tamoxifen at P5 and P6. (J) Liver sections of 12-month-old *Pkhd1*^*CreER/fl*^;*ZS-Green* rat induced with corn oil or tamoxifen at P5 and P6. (K) Immuno-fluorescence with anti-CK19 antibody (red) on liver sections of 12-month-old *Pkhd1*^*CreER/fl*^;*ZS-Green* rat and counterstained with DAPI induced with corn oil or tamoxifen at P5 and P6. (L) Mathematic formula of cyst size based on cell expansion and cell doubling time. (M) Plot a graph of cyst size by time based on the doubling time of 20 days and 30 days from a single cell. The dots on the right represent the measured diameter of liver cysts of 12-month-old *Pkhd1*^*CreER/fl*^;*Zs-Green* rats induced with tamoxifen at P5 and P6 (Figure S9H). Error bars represent mean ± s.e.m, ∗*p* < 0.05, ∗∗*p* < 0.01, and ∗∗∗*p* < 0.001 by Student’s *t* test (C and E). Scale bars, 2 mm (B); 1 mm (D); 200 μm (F, G, I, and K); 800 μm (H and J).

To perform mosaic analysis, *Pkhd1*^*CreER*^ rats were crossed with *Pkhd1*^*fl*^ and *ZsGreen* reporter to generate *Pkhd1*^*CreER/fl*^;*ZsGreen* animals. Tamoxifen induction at P5 and P6, sparse recombination converted *Pkhd1*^*CreER/fl*^ cells to *Pkhd1*^*CreER/-*^ (i.e., *Pkhd1*^−/−^) and activating ZsGreen expression (Figures 6A and S8). Control rats received corn oil only. At 1 year, tamoxifen-induced animals developed liver cysts, whereas corn oil-injected controls did not show any liver or kidney pathology (Figures 6B–6E and S9).

Mosaic recombination in the kidney did not result in cyst formation (Figures 6B and 6C). This is likely due to low recombination efficiency and the rarity of targeting to cells in the cystogenic tubule in the mosaic setting. Even in germline *Pkhd1*^−/−^ or *Pkhd1*^*CreER/CreER*^ rats, renal cysts developed in only a limited number of tubules (Figures S4 and S6D). In contrast, tamoxifen-induced *Pkhd1*^*CreER/fl*^ rats developed Zs-Green+ cysts in the liver by 12 months (Figures 6D, 6E, and S9H). At 1 month, individual ZSGreen+ cells were observed in renal tubules and bile ducts without morphological abnormalities (Figures 6F and 6G). By 12 months, extensive ZsGreen+ cysts were present in the liver, but not in the kidney, indicating robust expansion of *Pkhd1*^*CreER/-*^ cells marked by ZsGreen (Figures 6H–6K). We next assessed proliferation within these cysts. EdU labeling 3 h before sacrifice revealed that ∼1% of ZsGreen+ cyst-lining cells in *Pkhd1*^*CreER/fl*^ livers were EdU+, compared to ∼0% in uninduced controls (Figures S10A and S10B). These findings demonstrate that recessive loss of *Pkhd1* in individual cholangiocytes is sufficient to form cysts via expansion.

### Modeling proliferation dynamics in biliary cystic epithelia

Loss of FPC in a single cell, or a few cells, is sufficient to form liver cysts. To determine whether cell expansion alone explains cyst size, we developed a mathematical model of biliary epithelial cell proliferative dynamics. This model evaluates whether cell division under idealized conditions can recapitulate cyst growth kinetics or additional biological mechanisms are required.

Tubule cells undergo expansion in the process of cyst initiation and progression. These cells collectively form an approximately spherical epithelial layer, such that the sum of their apical surface areas corresponds to the total cyst surface area. This geometric relationship allows cyst surface area to be translated into lumen diameter, providing a quantitative link between cellular growth dynamics and cyst size over time.

Assuming a constant proliferation rate, no cell cycle exit, and a fixed ***dt***, the number of cyst-lining epithelial cells at time ***t*** is given by:

$$N_{\left(t\right)}=N_{\left(o\right)}\times 2^{\left(t/dt\right)}$$

where N_(o)_ is the initial number of cystogenic cells.

For a spherical cyst with idealized hexagonal packing (each cell surface area ≈64.95 μm^2^, derived from a 10 μm long diagonal), the cyst surface area becomes:

$$\text{Surface area}=N_{\left(0\right)}\times 2^{\left(t/dt\right)}\times 64.95$$

The diameter d of the sphere is then:

$$d=\sqrt{\frac{N_{\left(0\right)}\cdot 2^{\frac{t}{dt}}\cdot 64.95}{\pi }}$$

Using 3D reconstruction of *Brainbow* mice in the ARPKD model, we observed patches of fluorescently labeled cells within cysts traced from P28 to P90 (Figure 5D; Video S2). The number of cells per patch was approximately 4–8. An 8-cell clone corresponds to three cell doublings over 60 days (dt ≈ 20 days), while a 4-cell clone corresponds to two doublings (dt ≈ 30 days). Plotting cyst diameter over time revealed exponential growth (Figures 6L and 6M). Measurement from 1-year-old *Pkhd1*^*CreER/fl*^;*ZS-Green* rats induced at P5 yielded cyst diameters ranging from 0.089 mm to 4.346 mm (Figure S9H), with most values aligning closely with model predications (Figure 6M). These findings indicate that cell proliferation alone can account for biliary cyst growth to the observed scale.

However, biological reality introduces heterogeneity. Variations in cell cycle timing, local microenvironment, and paracrine signaling can produce disproportionate contributions from specific clones. For example, this model appears inapplicable to kidney cystogenesis. ZsGreen lineage tracing in renal cysts revealed single clones spanning ∼20 contiguous cells from P5 to P30 (Figure 2D), which in 3D reconstructions could represent hundreds of cells. In contrast, cyst progression slowed significantly during the subsequent period (Figure S4). Collectively, these data indicate that renal cysts undergo rapid, heterogeneous proliferation during the early stages. Short ***dt*** and larger initial cells (large ***N***_***(0)***_) enable millimeter-sized cysts within weeks.

Additional factors further modulate growth, epithelial cell shape transition from columnar to flattened can expand surface area, while apoptosis may remove subsets of cyst-lining cells and reduce overall size. Therefore, while our mathematical model supports that cell expansion is sufficient to explain cyst formation under idealized conditions, accurate *in vivo* predictions require incorporating biological variabilities and micro-environmental influences.

### Transcriptomic analysis identifies cell cycle gene upregulation in *Pkhd1*-deficient liver

To investigate pro-proliferative pathways suppressed by FPC, we performed transcriptomic profiling on early-stage cystic livers from *Pkhd1*^−/−^ and WT rats (Figures S11A–S11E). Differential gene expression analysis revealed 896 upregulated and 194 downregulated genes in the cystic livers (Figures S11A and S11B; Table S1). KEGG pathway enrichment revealed significant upregulation of pathways involved in cell cycle regulation, cell-extracellular matrix interactions, cell adhesion, and inflammatory signaling (Figures S11C–S11E).

RT-qPCR confirmed the upregulation of key cell cycle genes, including *Cdk1*, *Ccna2* (Cyclin A2), *Ccnb1* (Cyclin B1), *Cdkn2c*, *Plk1*, *Espl1*, *Mad2l1*, *Mcm3*, and *Bub1b* (Figure S11F). We validated anti-*Cdk1* and anti-*Ccna2* antibodies (Figures S12A and S12B), and the western blot revealed the upregulation of Cdk1 protein, with a moderate increase observed at P45 and strong elevation at 12 months (Figure S12C). Immunofluorescence revealed weak Cdk1 expression in WT bile ducts, whereas strong and homogeneous Cdk1 expression was observed in cyst-lining cells and surrounding tissues of *Pkhd1*^−/−^ livers (Figure S12D). Similar patterns were observed in *Pkhd1*^−/−^ mice (Figures S12E and S12F). For Ccna2, western blot confirmed increased expression at both P45 and 12 months (Figure S12G), immunostaining indicated that Ccna2 enrichment was primarily in surrounding stromal/parenchymal cells rather than CK19 positive cyst lining cells (Figure S12H), suggesting distinct proliferative contributions from stromal tissues. Therefore, in *Pkhd1* mutant livers, Cdk1 expression increases in biliary cysts and surrounding tissues, while Ccna2 expression is elevated specifically in stromal cells.

### Pharmacological inhibition of Cdk1 slows fibrocystic liver disease in *Pkhd1*^−/−^ mice

**Figure 7:**
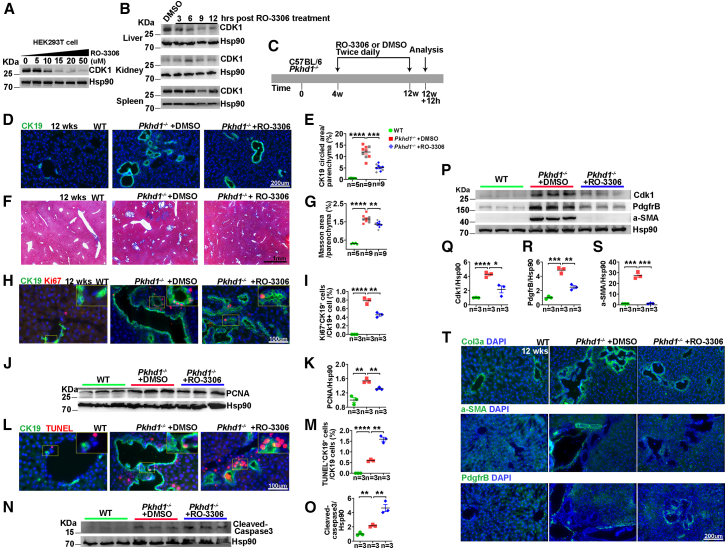
Pharmacological inhibition of Cdk1 with RO-3306 slows fibrocystic liver disease in *Pkhd1*^−/−^ mice (A) Expression of Cdk1 in HEK293T cells treated with different concentrations of RO-3306 via western blot. (B) Cdk1 expression in the liver, kidney, and spleen of mice treated with DMSO or RO-3306 via western blot. The organs were harvested at 3, 6, 9, and 12 h after the administration of four-week-old mice intraperitoneally injected with RO-3306 at a dosage of 20 mg/kg. (C) Schematic drawing of the treatment of *Pkhd1* mutant mice with DMSO or RO-3306. (D and E) Immuno-fluorescence with anti-Ck19 antibody on liver sections (D) and aggregated data of cystic index defined by Ck19 circled area per parenchyma captured (E) of WT (*n* = 5), *Pkhd1*^−/−^ mutant treated with DMSO (*n* = 9, male: symbol with color; female: symbol with gray), and *Pkhd1* mutant mice treated with RO-3306 (*n* = 9, male: symbol with color; female: symbol with gray) from P28 to P90. (F and G) Masson-Trichrome staining of liver sections (F) and aggregated data of the ratio of Masson staining positive area in the parenchyma captured (G) of the WT (*n* = 5), *Pkhd1*^−/−^ mutant treated with DMSO (*n* = 9, male: symbol with color; female: symbol with gray), and *Pkhd1* mutant mice treated with RO-3306 (*n* = 9, male: symbol with color; female: symbol with gray) from P28 to P90. (H and I) Immuno-fluorescence of anti-CK19 (green) and anti-Ki67 (red) (H) and aggregated data of the ratio of Ki67+Ck19+ cells in CK19+ cells (I) on the liver sections of WT (*n* = 3), *Pkhd1*^−/−^ mice treated with DMSO (*n* = 3), and *Pkhd1*^−/−^ mice treated with RO-3306 (*n* = 3). (J and K) Analyzing the expression of PCNA (J) via western blot and aggregated data of the densitometric ratios of PCNA to Hsp90 (K) in WT (*n* = 3), *Pkhd1*^−/−^ mice treated with DMSO (*n* = 3), and *Pkhd1*^−/−^ mice treated with RO-3306 (*n* = 3). (L and M) TUNEL (red) staining counter stained with anti-CK19 antibody (L) on the liver sections and aggregated data of the ratio of TUNEL+ cells within in CK19+ cells (M) on the liver sections of WT (*n* = 3), *Pkhd1*^−/−^ mice treated with DMSO (*n* = 3), and *Pkhd1*^−/−^ mice treated with RO-3306 (*n* = 3). (N and O) Analysis of the expression of cleaved-Caspase 3 (N) and Hsp90 via western blot and the densitometric ratios of cleaved-Caspase 3 to Hsp90 (O), which were normalized to the ratio of the WT group. (P) Analyzing the expression of CDK1, α-SMA, and PDGFβ in WT (*n* = 3), *Pkhd1*^−/−^ treated with DMSO (*n* = 3), and *Pkhd1*^−/−^ treated with RO-3306 (*n* = 3) livers via western blot. (Q, R, and S) Densitometric ratios of Cdk1 (Q), PDGFRβ (R), and α-SMA (S) to Hsp90 for in (P), which were normalized to the ratio of the WT group. (T) Immuno-fluorescence of anti-COL3a, anti-α-SMA, and anti-PDGFβ antibodies on the liver sections of WT, *Pkhd1*^−/−^ mice treated with DMSO, and *Pkhd1*^−/−^ mice treated with RO-3306. Error bars represent mean ± s.e.m, ∗*p* < 0.05, ∗∗*p* < 0.01, and ∗∗∗*p* < 0.001 by two-way ANOVA. Scale bars, 200 μm (D and T); 1 mm (F); 100 μm (H and L).

We investigate whether inhibiting cell cycle progression could slow cyst progression by targeting Cdk1. *Pkhd1*^−/−^ mice were treated with RO-3306, a potent ATP-competitive Cdk1 inhibitor. RO-3306 reduces Cdk1 protein steady state in tumors and cell lines.^23^^,^^24^ RO-3306 could effectively inhibit Cdk1 protein expression in HEK293T cells (Figure 7A) and hepatic Cdk1 levels for up to 12 h after a single injection, without affecting the kidney or spleen (Figure 7B).

Based on this profile, we administered RO-3306 or vehicle every 12 h from 4 to 12 weeks of age via intraperitoneal injection (Figure 7C). To evaluate the therapeutic impact on liver pathology, we quantified the CK19+ cystic area as a measure of cyst progression and the Masson-stained area as an indicator of the extent of fibrosis. We found that RO-3306 treatment significantly reduced both the CK19+ cystic area (Figures 7D and 7E) and the Masson-stained area (Figures 7F and 7G), indicating that RO-3306 attenuates the pathological progression of fibrocystic liver disease. We next examined the cellular changes underlying the reduced cyst burden. RO-3306 treatment decreased cholangiocytes proliferation, evidenced by reduced the percentage of Ki67+ cells among CK19+ cyst-lining cells (Figures 7H and 7I) and reduced PCNA levels (Figures 7J and 7K). In parallel, RO-3306 treatment increased cyst lining cell apoptosis, assessed by TUNEL assay and cleaved caspase-3 levels (Figures 7L–7O). Western blotting confirmed that the expression of Cdk1 was downregulated by RO-3306 treatment (Figures 7P and 7Q). Consistent with the reduced fibrosis observed in Masson’s trichrome staining, the expression of key fibrotic markers, including α-SMA, PDGFRβ, and Collagen III, was also substantially decreased via immunofluorescence and western blot (Figures 7P–7T). Collectively, these data indicate that Cdk1 is a key driver of cystic proliferation and fibrotic remodeling in *Pkhd1-*deficient livers, and that its pharmacological inhibition slows fibrocystic liver disease, providing proof-of-principle that targeting pro-proliferative signaling downstream of FPC loss could be an effective therapeutic strategy for ARPKD.

## Discussion

In this study, we developed lineage tracing analysis tools in both rats and mice to investigate renal tubule cell and cholangiocyte behavior and contribution to cyst formation in ARPKD, and to infer the epithelial dynamics of cystogenesis in the kidney and liver. Using single-color lineage tracing in rats and multicolor clonal analysis in mice, we found that the individual epithelial cells undergo expansion, consistently accompanying the development of cystic structures from renal tubules and bile ducts. Individual cholangiocytes with recessive loss of *Pkhd1* are sufficient to form cysts via expansion in genetic mosaic analysis. From the single cell behavior and contribution, we infer the collective behavior of a cyst forming tubule and bile duct composed of multiple cells, cyst maintains monolayer of cyst lining cells and the cyst lining cells collectively expand laterally, and contributes to continuously growth of cyst size. Transcriptomic profiling of early-stage polycystic liver tissue revealed the upregulation of multiple cell cycle genes, including *Cdk1*, whose protein product is expressed strongly in cyst-lining cells. Pharmacological inhibition of Cdk1 with RO-3306 slows cyst progression and attenuates fibrosis, indicating that Cdk1 is a key driver of cyst progression in this context.

Our results suggest that FPC acts to suppress a pro-proliferative program and maintain tubule diameter. When FPC levels fall below a critical threshold, this inhibition is attenuated or lost, unleashing proliferative signaling that drives epithelial expansion and cyst formation. How this single-pass TM protein exerts its inhibitory role remains unclear, underscoring the need to identify downstream pathways directly regulated by FPC. The intra-cellular domain of FPC appears dispensable for tubule structure maintenance and cyst suppression in mice, as the deletion of exon 67, which removes the intra-cellular domain, does not result in cyst formation.^6^ Despite this, the intra-cellular domain harbors motifs for ciliary, nuclear, and mitochondrial targeting,^25^^,^^26^^,^^27^ which may regulate processes like metabolism.^28^

Lineage tracing enabled us to re-examine the contribution of the disruption of OCD, a pivotal PCP-dependent mechanism driving tubule elongation along the longitudinal axis, to cyst initiation. In the collecting duct of rats, the medullary collecting ductnbsp;typically follows the longitudinal axis of the nephron, with only a small proportion exhibiting transverse division. In *Pkhd1*^*CreER/CreER*^;*ZsGreen* rats, there is an increase in the proportion of transverse division revealed by both lineage tracing and examination of division axis of the collecting duct cells; therefore, loss of FPC disrupts OCD, similar to observations in *Pkhd1*^*del4*^ mice.^5^^,^^12^ These findings suggest that loss of FPC-generated OCD alone is insufficient to initiate cyst formation.^12^ However, we do not exclude the possibility that the more severe and embryonic-onset cystic kidney phenotype observed in humans, PCP defects may contribute more substantially to disease progression, potentially in conjunction with elevated proliferation.

Using our knock-in *Pkhd1*^*CreER*^ rats, we found that *Pkhd1* expression is broader than previously appreciated. Reporter activity revealed expression in the collecting duct, thick ascending limb, distal convoluted tubule, and proximal tubule. Consistently, immunofluorescence with anti-HA antibody to the Pkhd1-HA protein expressed by the *Pkhd1*^*HA*^ knock-in mice reveals that endogenous FPC-HA is expressed in the proximal tubule transiently, in addition to its well-documented localization to the collecting duct.^11^ Thus, our findings indicate that ARPKD cysts originate from multiple nephron segments, challenging the long-standing assumption that only collecting ducts are involved. Based on histological comparison, the disease severity in our *Pkhd1*^*CreER/CreER*^ rats is less severe than in *PCK* rats.^10^^,^^29^ Our preliminary explanation is that *Pkhd1*^*CreER*^ disrupts *Pkhd1* function, and it also expresses CreER protein in the cytoplasm of epithelial cells in the distal nephron, which adds new variation to the system and may confer some protection against cyst growth in an unidentified way.

Our *Pkhd1* mutant rats develop extensive biliary cyst formation in the liver; however, both *Pkhd1* mutant and *PCK* rat models demonstrate that rodent ARPKD models fail to develop severe renal cystic disease characteristic of human ARPKD.^30^ Similarly, mouse ARPKD models typically exhibit tubular dilation rather than overt renal cyst formation.^5^^,^^11^^,^^31^ Collectively, these findings indicate that rodent genetic models do not fully recapitulate the renal pathology observed in human ARPKD.

We acknowledge that our mathematical model is intentionally simplified. It explicitly links epithelial expansion at the cellular scale to cyst growth at the tissue scale within a quantitative framework, integrating experimentally observed processes into a unified mathematical structure. By consolidating existing biological insights, the model provides a conceptual foundation that can support future hypothesis-driven extensions and experimental testing. While cystogenesis is influenced by multiple additional factors that may either promote or restrain growth, incorporating all such influences as independent parameters would substantially increase model complexity. In this context, the current level of abstraction allows the model to isolate and clarify the contribution of core cellular behaviors, while remaining amenable to future refinement as additional mechanisms are defined.

Our findings also have broader implications for cystic disease. Elevated proliferation is a shared feature of both ARPKD and ADPKD,^32^^,^^33^ and Cdk1 has been shown to drive renal cyst progression in ADPKD genetic models as well.^34^ Moreover, our study reveals that targeting Cdk1 not only slows cyst growth but also reduces liver fibrosis, reinforcing the tight interconnection between epithelial proliferation and fibrotic remodeling in ARPKD. These results highlight the function of Cdk1 and other cell cycle regulators for cyst formation.

The reason why RO-3306 affects the steady-state of Cdk1 protein differently in the liver compared to the kidney and spleen is unknown. We hypothesize that the pharmacological dynamics of RO-3306 lead to its different distribution among organs. Specifically, as the liver is the primary site for the modification and detoxification of xenobiotics, the local concentration of the active compound may be higher. This elevated, temporary concentration in the liver could target a greater proportion of Cdk1, thereby disrupting its steady state more significantly.

Although RO-3306 has been shown to slow fibrocystic liver disease in a mouse model, we do not recommend its use in patients with ARPKD, who are typically children undergoing continuous growth and development. RO-3306 exerts its effect by slowing proliferation and promoting apoptosis, thus serving as a proof of principle that targeting the pro-proliferative pathway(s) involved in cyst progression can effectively limit cell proliferation and disease advancement. Therefore, it is imperative to identify the specific pro-proliferative signals driving cystogenesis, as these may represent promising therapeutic targets.

In summary, by tracing individual renal tubule and bile duct cells during cyst formation, we found that cystogenic epithelial cells expand significantly to drive cyst formation and growth in ARPKD ortholog models. This suggests that the loss of FPC results in the activation of a pro-proliferative signal(s), with Cdk1 emerging as a key downstream effector. These insights clarify the cellular basis of ARPKD progression and provide a framework for identifying FPC-suppressed pro-proliferative signaling pathways, which may serve as safe and effective therapeutic targets.

### Limitations of the study

One limitation of our study is that bulk liver transcriptomics may dilute bile duct-specific transcriptional changes, since bile ducts constitute only a small fraction of liver tissue. Future single-cell transcriptomic profiling will be needed to resolve cholangiocyte-specific alterations. Nonetheless, our bulk liver transcriptomic profiling revealed both cell-autonomous and non-cell-autonomous transcriptional changes in fibrocystic liver disease. Notably, *Cdk1* expression was upregulated in cyst-lining epithelial cells and adjacent fibrotic tissues, whereas *Ccna2* expression was enriched in the surrounding fibrotic tissue, suggesting active stromal-epithelial crosstalk.

## Resource availability

### Lead contact

Requests for further information and resources should be directed to and will be fulfilled by the lead contact, Ming Ma (skymaming@swu.edu.cn).

### Materials availability

There are restrictions on the availability of our rat strain because of the lack of an external centralized repository for its distribution and our need to maintain the stock. We are glad to share them with reasonable compensation upon request for their processing and shipping. All unique/stable reagents generated in this study are available from the lead contact with a completed materials transfer agreement.

### Data and code availability

• RNA-seq data have been deposited at the National Center for Biotechnology Information Sequence Read Archive (SRA): PRJNA1065508 and are publicly available as of the date of publication.
• Original western blot images have been deposited at Mendeley at [https://doi.org/10.17632/hcyv8nb8b8.1] and are publicly available as of the date of publication. Microscopy data reported in this paper will be shared by the lead contact upon request.
• This paper does not report original code.
• Any additional information required to reanalyze the data reported in this paper is available from the lead contact upon request.

## Acknowledgments

The authors are grateful to Dr. Stefan Somlo, Dr. Feng Qian, and Dr. Sudipto Roy for their critical reading the manuscript and valuable advice. This work was supported by grants from the 10.13039/501100012166National Key Research and Development Program of China (2019YFA0802704), the 10.13039/501100001809National Natural Science Foundation of China (31771620), Research Startup Fund of 10.13039/501100006250Southwest University (SWU117064), and NHC Key Laboratory of Birth Defect for Research and Prevention (Hunan Provincial Maternal and Child Health Care Hospital) no. KF2021004, and the Open Project of Hainan Provincial Key Laboratory for Human Reproductive Medicine and Genetic Research to M. M.

## Author contributions

Conceptualization, S.L. and M.M.; methodology, S.L. and M.M.; investigation, S.L., X.C., J.T., H.T., and Z.L.; writing – original draft, S.L. and M.M.; writing – review and editing, S.L. and M.M.; funding acquisition, Y.P., X.M., R.D., B.Z., and X.L.; resources, L.L. and L.L.; supervision, M.M.

## Declaration of interests

The authors declare no competing interests.

## Declaration of generative AI and AI-assisted technologies in the writing process

During the preparation of this work, the authors did not use any generative AI or AI-assisted technologies.

## STAR★Methods

### Key resources table

**Table:** 

REAGENT or RESOURCE	SOURCE	IDENTIFIER
**Antibodies**		

rabbit-*anti*-Cdk1 antibody	Proteintech	Cat#19532-1-AP; RRID: AB_10638617
rabbit-*anti*-CCNA2 antibody	Proteintech	Cat#18202-1-AP; RRID: AB_10597084
mouse-*anti*-Ki67 antibody	Cell Signaling Technology	Cat#9449T
rabbit anti-alpha-SMA antibody	Affinity	Cat#AF1032; RRID:AB_2835329
rabbit-*anti*-Collagen Type III	Proteintech	Cat#22734-1-AP; RRID:AB_2879158
rabbit-*anti*- PDGFR beta antibody	Proteintech	Cat#13449-1-AP; RRID:AB_2162644
rabbit-*anti*-Laminin gamma 1	Abcam	Cat#ab233389; RRID:AB_3076401
mouse anti-Aquaporin 2 antibody	Santa Cruz	Cat#SC-515770; RRID:AB_2810957
mouse anti-Megalin/LRP2 antibody	Santa Cruz	Cat#sc-515750
rabbit-*anti*-Uromodulin(THP) antibody	Proteintech	Cat#11911-1-AP
Anti-UMOD antibody	Abcam	Cat#Ab207170; RRID:AB_10695625
Calbindin-D28k Polyclonal antibody	Proteintech	Cat#14479-1-AP; RRID:AB_2228318
mouse-*anti*-CK19 antibody	Abcam	Cat#ab220193; RRID:AB_2814863
rabbit-*anti*-CK19 antibody	Abcam	Cat#Ab133496; RRID:AB_11155282
rabbit-*anti*-Cleaved Casepase3 antibody	BD Bioscience	Cat#559565; RRID:AB_397274
mouse-*anti*-PCNA antibody	Proteintech	Cat#60097-1-1g; RRID:AB_2236728
rabbit-*anti*-Hsp90 antibody	Proteintech	Cat#13171-1-AP; RRID:AB_2120924
goat anti-rabbit IgG H&L/HRP	Bioss	Cat#bs-0295G-HRP; RRID:AB_10923693
goat anti-Mouse IgG (H + L) Cross-Adsorbed Secondary Antibody, Alexa Fluor™ 594	Invitrogen	Cat#A11005; RRID:AB_141372
goat anti-Rabbit IgG (H + L) Cross-Adsorbed Secondary Antibody, Alexa Fluor™ 488	Invitrogen	Cat#A11008; RRID:AB_143165
donkey anti-Rabbit IgG (H + L) Highly Cross-Adsorbed Secondary Antibody, Alexa Fluor™ Plus 594	Invitrogen	Cat#A32754; RRID:AB_2762827
donkey anti-Rabbit IgG (H + L) Highly Cross-Adsorbed Secondary Antibody, Alexa Fluor™ 488	Invitrogen	Cat#A21206; RRID:AB_2535792

**Chemicals, peptides, and recombinant proteins**		

EdU(5-ethynyl-2' -deoxyuridine)	Beyotime	ST067-1g
4-OH tamoxifen	MCE	HY-16950
Tamoxifen	Macklin	T832955
RO-3306	MCE	HY-12529

**Critical commercial assays**		

NovoStart®SYBR qPCR SuperMix plus	Novoprotein	E096
BeyoClick™ EdU-594 Cell Proliferation Assay Kit	Beyotime	C0078S
TUNEL Apoptosis Detection Kit (FITC)	Yeasen	40306ES20
QuantiChrom^TM^ Urea Assay Kit	BioAssay Systems	DIUR-500
QuantiChrom^TM^ Creatinine Assay Kit	BioAssay Systems	DICT-500

**Deposited data**		

Original western blot data	This paper	https://doi.org/10.17632/hcyv8nb8b8.1
Bulk-RNA seq data	This paper	PRJNA1065508

**Experimental models: Cell lines**		

Human: HEK293T cells	ATCC	CRL-3216
Rat: Primary renal cells	This paper	N/A

**Experimental models: Organisms/strains**		

Rat: Sprague-Dawley: *Pkhd1*^*CreER*^	This paper	Cyagen
Rat: Sprague-Dawley: *Pkhd1*^*fl*^	This paper	Cyagen
Rat: Sprague-Dawley: Tg:CAG-Ncre 81Jmsk rats	Rat Resource & Research Center	RRRC#: 00301
Rat: Sprague-Dawley: F344-Tg:CAG-loxP-STOP-loxP-ZsGreen 561Bryd rats	Rat Resource & Research Center	RRRC#: 00797
Mouse: C57/B6: *Pkhd1*^*del4-5/del4-5*^ (*Pkhd1*^−/−^)	This paper	Cyagen
Mouse: B6.129P2-Gt(ROSA)26Sor^tm1(CAG−Brainbow2.1)Cle^/J	Jackson Laboratory	JAX: 017492
Mouse: Krt19^tm1(cre/ERT)Ggu^/J	Jackson Laboratory	JAX: 026925

**Oligonucleotides**		

Primers for qPCR, see Table S2	This paper	N/A
Primers for rat genotyping, see Rat strains and procedures	This paper	N/A
Primers for mouse genotyping, see Mouse strains and procedures	This paper	N/A

**Recombinant DNA**		

Plasmid: pcDNA3.1(+)-Cdk1-HA	This paper	N/A
Plasmid: pcDNA3.1 (+)-Ccna2-GFP	This paper	N/A

**Software and algorithms**		

ImageJ	NIH	https://imagej.nih.gov/ij/
ZEN	Zeiss	https://www.zeiss.com.cn/microscopy/products/software/zeiss-zen.html
Imaris	Oxford Instruments	https://imaris.oxinst.com/
Rstudio	R	https://www.r-project.org/
Prism 10	GraphPad	https://www.graphpad.com
Cellsens Standard	Olympus	https://evidentscientific.com/en/products/software/cellsens
NIS-Elements BR	Nikon	https://www.microscope.healthcare.nikon.com/en_AOM/products/software/nis-elements/nis-elements-basic-research

### Experimental model and study participant details

#### Rat and mouse husbandry

All animals were kept in Specific Pathogen Free (SPF) facility with 12h light/dark cycle at 22 ± 2 °C and 50±10% relative humidity. All experimental procedures were approved by the Institutional Animal Care and Use Committee of Southwest University (IACUC No. Approved: IACUC-20251110-01). The phenotypes of renal and hepatic cysts are not sex-dependent. Unless otherwise stated, all experimental animals were included based on age-matched genotypes, irrespective of sex or cage location. Euthanasia was performed using an R550 Multi-output Animal Anesthesia Machine (RWD).

#### Rat strains and procedures

*Pkhd1*^*CreER*^ rat was generated by CRISPR/Cas9-mediated insertion of a *CreERT2-SV40* late pA cassette downstream of the ATG start codon, replacing exons 2-5. Cas9, gRNA and the donor vector were co-injected into one-cell stage Sprague-Dawley embryos (Cyagen). gRNA sequences are as follows: 5’ CGCCAGGCAGGCATCACATCAGG’3; 5’TGACTTCACCACTTATTGCCTGG’3.

*Pkhd1*^*flox*^ rats were generated by CRISPR/Cas9 insertion of *loxP* sites flanking exons. Cas9, gRNA and donor vector were co-injected into one-cell stage SD embryos (Cyagen). gRNA sequences are as follows: 5’ GACCCGCACACAAGGCCGACTGG ’3; 5’ CTCTGAAGGAGCGGCCCGAGAGG ’3.

The Wistar-Tg:*CAG-Ncre* 81Jmsk rats (RRRC#: 00301) and F344-Tg:*CAG-loxP-STOP-loxP-ZsGreen* 561Bryd rats (RRRC#: 00797) were ordered from the Rat Resource & Research Center. The following primers were used for genotyping: G-F: 5’ ATGTGTGGATTTGGTCGGGC’3; G-R: 5’ GCAGCCATTTGTCGCCAAGA’3; C-F:5’GGACCGGTTTCTCTCATTT’3; C-R:5’CCACTTGATGGGTTAACTTG’3; R2: 5’CGAACCTCATCACTCGTTGC’3.

The *Pkhd1* null allele was obtained by crossing *Pkhd1*^*flox*^ and *CAG-Ncre* Rats. The following primers were used for genotyping: UF: 5’ GCTCATCCAAGATGACTGTCC’3;UR: 5’ TGAAGGCCTGATTCAGCCTGCA ’3; dR:5’ATGGCCACTGCAAGTTACAC’3; F1: 5’ TTGAAGTTGGAAATGCTGTT ’3; R1: 5’ TCTCTGGGCTTGGGCTCGGA ’3.

#### Mouse strains and procedures

*Pkhd1*^*-/-*^ mice (stock S-KO-07253) were ordered from Cyagen. *Rosa*^*Brainbow*^ (stock #: 017492), *CAG*^*CreERT2*^ (stock #: 004682), and *Ck19*^*CreER*^ (stock #: 026925) were ordered from Jackson Laboratory.

Primers F(5`-TGGGCTTTCCTGAATTTGGGTG-3`), R1 (5`-CAAGGAAACT GTTCTCA GCCTTG-3`) and R2(5`-GCCACAAGAGGGCAATGTAAG-3`) were used for *Pkhd1*^*-/-*^ mice genotyping. Primers 11341(5′-GAATTAATTCCGGTATAA CTTCG-3′), oIMR8545 (5′- AAAGTCGCTCTGAGTTGTTAT-3′), and oIMR8916 (5′- CCAGATGACTACCTATCCTC-3′) were used for *Rosa*^*Brainbow*^ genotyping. Primers M180F(5′-GCTAACCATGTTCATGCCTTC-3′), and M180R (5′-AGGCAAATTTTGGTGTACGG-3`) were used for *CAG*^*CreERT2*^ mice genotyping. Primers CK19-F (5′-GCAGAATCGCCAGGAATTGACC-3′), and CK19-R (5′-GTTCTTGCGAACCTCATCACTC-3′) were used for *CK19*^*CreER*^ mice genotyping. *Rosa*^*mTmG*^ genotyping. Primer 12177: CTTTAAGCCTGCCCAGAAGA Primer 30297: TAGAGCTTGCGGAACCCTTC primer 30298: AGGGAGCTGCAGTGGAGTAG.

#### Generating experimental animals and genotyping

The schematic diagrams of the mating strategy for this study are shown in Figure S14. For genotyping, genomic DNA or cDNA was extracted and analyzed via PCR using the specific primers described previously. The PCR procedure is as follows: 98°C for 3 min; 98°C for 10 s; 60°C (variable) for 10 s; 72°C for 15s; 72°C for 5 min.

### Method details

#### Tamoxifen administration

Tamoxifen (Macklin, T832955) was dissolved in corn oil (Aladdin, C116025) at 20 mg/ml. *CK19*^*CreER*^*;Rosa*^*Brainbow/+*^ mice at postnatal 28 (P28) received a single intraperitoneal injection of tamoxifen with 200 mg/kg of body weight. *Pkhd1*^*CreER/CreER*^ and *Pkhd1*^*CreER/+*^ rats were injected intraperitoneally with a single dose of tamoxifen with 200 mg/kg at P5. *Pkhd1*^*CreER/fl*^ rats were injected intraperitoneally with two doses of tamoxifen (200 mg/kg) at P5 and P6 or corn oil as control. Animals were assigned to tamoxifen or vehicle groups irrespective of gender.

#### Primary kidney cell culture and induction with 4OH-tamoxifen

*Pkhd1*^*CreER/+*^*;ZsGreen* rats at postnatal day 3 (P3) were euthanized, and kidneys were harvested and digested in DMEM/F12 medium containing 1 mg/ml collagenase (Yuanye, S10054) and 1 mg/ml hyaluronidase (Yuanye, S10060) at 37°C for 2 hours. The enzymatic reaction was stopped with FBS. Cells were then washed with DMEM/F12 medium (Gibco, C11330500BT) containing 10% FBS and passed through a 40 μm cell strainer. The resulting cell suspension was cultured in DMEM/F12 with 10% FBS, supplemented with 4-OH tamoxifen (MCE, HY-16950) (1, 5, 10 or 20 μM) or vehicle, for 16 hours. Cell cultures were maintained in 5% CO2 at 37°C. Prior to imaging on a Nikon DS-Qi2, cells were stained with Hoechst.

#### Labelled cell clone frequency analysis

For clonal frequency analysis in the kidneys of *ZSGreen* rats, all ZS+ cysts in *Pkhd1*^*CreER/CreER*^ rats were counted. The number of contiguous fluorescent cells was determined by counting number of co-stained DAPI positive nuclei. Continuous ZS+ fluorescence was quantified in *Pkhd1*^*CreER/+*^ tubule (42 ZS+ stretches from 3 rats), *Pkhd1*^*CreER/CreER*^ non-dilated tubule (36 ZS+ stretches from 3 rats), and *Pkhd1*^*CreER/CreER*^ cyst (40 ZS+ stretches from 7 rats) at P30.

For liver clonal frequency analysis, ZS+ fluorescence in bile duct and cysts was randomly selected for quantification. The number of contiguous fluorescent cells was estimated based on the number of continuous ZS+ dots. Continuous ZS+ fluorescence was counted in *Pkhd1*^*CreER/+*^ cholangiocytes (44 ZS+ stretches from 3 rats) and *Pkhd1*^*CreER/CreER*^ cysts (47 ZSGreen+ stretches from 3 rats) at P90.

For *Rosa*^*Brainbow*^ mice, the number of cells per clone sharing the same fluorophores was recorded and categorized into clusters of 1 cell, 2 cells, 3 cells, 4 cells, 5-10 cells, and >10 cells. Clonal frequency was calculated using the formula:

$$Clone\;frequency\left(\%\right)=\left(N_{n}/N_{t}\right)\times 100\%$$

where *N*_*n*_ is the number of n-cell clones and *N*_*t*_ is the total clone count. For each animal, three to five randomly selected fields of view were analyzed from three animals of the same genotype.

#### Collecting duct cell division angle measurement

*Pkhd1*^*CreER/+*^;*ZSGreen* and *Pkhd1*^*CreER/CreER*^;*ZSGreen* rats were induced at P5 with a single intraperitoneal dose of tamoxifen (200 mg/kg). Rats were sacrificed at P15, and the coronal kidney sections (50 μm) were prepared and imaged using a Nikon DS-Qi2 microscope. The division angle was defined as the angle between the longitudinal axis of a labelled nephron (continuous stretch of ZSGreen+ cells) and the axis connecting two labeled cells at the end. For *Pkhd1*^*CreER/CreER*^;*ZSGreen* rats, 54 ZSGreen+ tubules division angles were measured from 5 rats. For *Pkhd1*^*CreER/+*^;*ZSGreen* rats, 52 ZSGreen tubules division angles were measured from 7 rats.

Measurement of mitotic angles refers to Fisher^9^ and Saburi.^35^ In brief, *Pkhd1*^*CreER/+*^ and *Pkhd1*^*CreER/CreER*^ rats were sacrificed at P10-P15, and the coronal kidney sections (50 μm) were prepared for Immunofluorescence with anti-Aqp2 (Santa Cruz, SC-515770) and anti-H3pS10 (Santa Cruz, SC-8656-R) antibodies. Then the sections were imaged with 1-μm-interval Z-steps (30-50 Z-Stack images) using Zeiss LSM 880 Laser scanning confocal microscope. The images were 3D-reconstructed using the ZEN and Imaris 9.0.1 software. The 3D reconstruction of Aqp2-positive cells defined the axial direction of the collecting duct lumen. The mitotic division angle is represented as the angle between consecutive p-H3-positive cells along the axial direction. For *Pkhd1*^*CreER/CreER*^ rats, 56 mitotic angles were measured from 5 rats. For *Pkhd1*^*CreER/+*^;*ZSGreen* rats, 55 mitotic angles were measured from 5 rats.

The Mann-Whitney U test was used for division angle comparison between *Pkhd1*^*CreER/+*^*;ZsGreen* and *Pkhd1*^*CreER/CreER*^*;ZsGreen* rats.

#### Histology

The left kidneys and the largest liver lobes were fixed in 4% paraformaldehyde (PFA). Hemolytoxin and Eosin (H&E) staining and Masson’s trichrome staining were performed by Servicebio.

#### Tissue sectioning

Tissues from euthanized rats or mice were fixed in 4% paraformaldehyde at 4°C overnight, followed by immersion in 30% sucrose (in PBS) overnight. Samples were then embedded in Tissue-Tek OCT (Sakura Finetek, 4583), and were rapidly frozen and stored in -80 °C. Cryosections were prepared at −20°C with thickness of 5-10 μm or 50 μm as required.

#### Blood urea nitrogen and serum creatinine measurement

Rat blood samples were collected via cardiac puncture into lithium Heparin tubes (Kangweisi, KWS). Serum was obtained by centrifuging at 6000 rpm for 5 minutes. Serum urea nitrogen was measured using the QuantiChrom^TM^ Urea Assay Kit (BioAssay Systems, DIUR-500), and creatinine was measured with QuantiChrom^TM^ Creatinine Assay Kit (BioAssay Systems, DICT-500).

#### Renal cystic index measurement

Hemolytoxin and eosin-stained kidney sections were scanned using an Olympus microscope. The cystic index was calculated as the percentage of the ratio of cystic area to the total kidney area measured with Cellsens Standard software (Olympus).

#### Biliary cystic index measurement

The left lobes of liver sections were fixed, and coronal sections were subjected to immuno-fluorescence staining with anti-CK19 antibody. The biliary cystic index was calculated as the ratio of the CK19 circled area to the total area using ImageJ. For each animal, three to five randomly selected fields of view were measured and averaged.

#### RO-3306 administration

*Pkhd1*^*-/-*^ mice were administered with RO-3306 (*n =* 9, 5 male and 4 female) or DMSO (*n =* 9, 5 male and 4 female) every 12 hours from 4 weeks to 12 weeks of age. The final dose was administered 12 hours before euthanasia. Sample size and power calculations were performed using STPLAN (ver. 4.5; University of Texas, MD Anderson Cancer Center). Calculations were based on the following: the cystic index (the average of the percentage of Ck19 circled area within randomly selected parenchyma area of the coronal section of the left lobe of the liver) of each *Pkhd1*^*-/-*^ mouse liver at P90 is 12.14% (s.d. of ± 3.08%) based on our empirical data. Our alternative hypothesis was that RO-3306 treatment to *Pkhd1*^*-/-*^ mice would result in a 50% decrease in cystic index to 6.07% (s.d. of ± 3.08%) with a significance level (α) of 0.05 (two sided). Under these expectations, five mice could achieve 90.1% power to detect the difference of 6.07% between the null hypothesis and the alternative hypothesis.

#### Reverse transcription and quantitative-PCR

Total RNA was extracted from rat kidneys and livers using Trizol reagent (Yeasen, 10606ES60). Complementary DNA (cDNA) was synthesized using GoScript™ Reverse Transcriptase (Promega, A5003). Quantitative PCR (qPCR) was performed with NovoStart®SYBR qPCR SuperMix plus (Novoprotein, E096) and analyzed by LightCycler® 96 System (Roche). Primers were designed by NCBI Primer-BLAST and are listed in Table S2.

#### Imaging

For *in vivo* fluorescence detection of *Rosa*^*Brainbow*^ mouse liver, samples were imaged using a Zeiss LSM 880 Laser scanning confocal microscope. Excitation wavelengths were 405 nm (CFP), 488 nm (GFP), 514 nm (YFP), and 561 nm (RFP). Confocal image acquisition and 3D reconstruction were processed with ZEN software. All other samples were imaged with Nikon DS-Qi2, and the images were analyzed by the NIS-Elements BR (version 5.10.00) software.

#### Proliferation assay

Experimental rats or mice were injected intraperitoneally with 50 mg/kg EdU (Beyotime, ST067-1g) 3 hours before euthanasia. Cell proliferation was assessed using the BeyoClick™ EdU-594 Cell Proliferation Assay Kit (Beyotime, C0078S), followed by immuno-fluorescence with anti-Aqp2 or anti-CK19 antibodies and counter stained with DAPI.

For kidneys, over 1,000 Aqp2 positive cells were counted for each wild type (WT) or mutant animal. For livers, over 800 CK19 positive cells were counted per mutant liver, and 80 to 200 CK19 positive cells were counted per WT liver. The proliferation rate was calculated as the number of EdU positive cells per 1000 Aqp2 (kidney) positive or CK19 (liver) positive cells.

#### Apoptosis assay

Apoptosis assay was performed using the TUNEL Apoptosis Detection Kit (FITC) (Yeasen, 40306ES20), followed by immuno-fluorescence staining with anti-Aqp2 or anti-CK19 antibodies and stained with DAPI. For mouse livers, over 800 Ck19 positive cells were counted in cystic regions of mutant liver sections, and 80-200 CK19 positive cells were counted per WT liver.

#### Generate mathematic model for cyst formation

Assuming constant proliferation rate and no cell cycle exit, and a fixed doubling time throughout cystogenesis, the number of cyst-lining epithelial cells over time (t) is modeled as:

$$N_{\left(t\right)=N_{\left(o\right)}\times {\;2}^{\left(t/dt\right)}}$$

Where N_(o)_ is the initial number of cystogenic cells and dt is the doubling time.

Using 3D reconstruction of *Rosa*^*Brainbow*^ mice in ARPKD model, we observed clonal patches of fluorescently labeled cells within cysts traced from P28 to P90 (Figure 5D; Video S2). The number of cells per clone was approximately 4-8, 8 cells would suggest three rounds of division over 60 days, implying a doubling time of 20 days. Thus:

$$N_{\left(t\right)=N_{\left(o\right)}\times {\;2}^{\left(t/20\right)}}$$

Assuming cystic cells originate at P5 and proliferate for 360 days, we estimate the final cell count:

$$N_{\left(360\right)}=N_{\left(0\right)}\times 2^{\left(360/20\right)}=N_{\left(0\right)}\times 262,144\;cells$$

Assuming spherical cysts and idealized hexagonal packing with each cell occupying a surface area of ∼64.95 μm^2^ (derived from a long diagonal of ∼10μm), the total surface area of a 262,144-cell cyst would be:

$$Surface\;area=262,144\times 64.95=∼17,026,252\;{\mu m}^{2}$$

Solving the diameter (d) of a sphere with this surface area:

$${\pi d}^{2}=17,026,252\;{\mu m}^{2}$$

d=2,328μm=2.328mm

This suggests that under ideal conditions, a cyst originating from a single cell could grow to over 2 mm in diameter within a year.

If the number of cells per clone was 4, suggests two rounds of division over 60 days, implying a doubling time of 30 days.

$$N_{\left(360\right)}=N_{\left(0\right)}\times 2^{\left(360/30\right)}=N_{\left(0\right)}\times 4,096\;cells$$

Assuming spherical cysts and idealized hexagonal packing with each cell occupying a surface area of ∼64.95 μm^2^ (derived from a long diagonal of ∼10μm), the total surface area of a 4,096-cell cyst would be

$$Surface\;area=4096\times 64.95=∼266,035\;{\mu m}^{2}$$

Solving the diameter (d) of a sphere with this surface area:

$${\pi d}^{2}=266,035$$

d=∼291μm=∼0.291mm

#### Immuno-fluorescence

Kidney and liver tissues were fixed in 4% paraformaldehyde (PFA), and immersed in 30% sucrose solution in PBS, and embedded in OCT medium (Sakura). Sections (5-10 um) were cut on a Leica Cryostat Microtome. For permeabilization, sections were incubated in 0.5% Sodium dodecyl sulfate (SDS) in PBS at room temperature for 5 minutes, followed by blocking with 10% goat serum containing 0.1% bovine serum albumin (BSA) for 1 hour. Sections were incubated with primary antibodies overnight at 4°C and were washed with PBS for 3 times 10 minutes each. The sections were incubated with secondary antibodies for 1 hour at room temperature and washed in PBS for 3 times, 10 minutes each. Sections were counterstained by 4',6-diamidino-2-phenylindole (DAPI). Sections were mounted in Anti-Fluorescence Quenching Agent (elabscience, E-IR-119).

The following antibodies and lectins were used: Rhodamine-Dolichos Biflorus Agglutinin (DBA) (1:50, Vectors Laboratories, RL-1032-2); rabbit-anti-Cdk1 antibody (1:200, Proteintech, 19532-1-AP); rabbit-anti-CCNA2 antibody (1:5000, Proteintech, 18202-1-AP); mouse-anti-Ki67 antibody (1:1000, Cell Signaling, 9449T); rabbit anti-alpha-SMA antibody (1:200, Affinity, AF1032); rabbit-anti-Collagen Type III (N-terminal) antibody (1:200, Proteintech, 22734-1-AP); rabbit-anti- PDGFR beta antibody (1:200, Proteintech, 13449-1-AP); rabbit-anti-Laminin gamma 1 (1:100, Abcam, ab233389); rabbit anti-Aquaporin 2 antibody (1:50, BOSTER, BA0649); mouse anti-Aquaporin 2 antibody (1:100, Santa Cruz, SC-515770); mouse anti-Megalin/LRP2 antibody (1:200, Santa Cruz, sc-515750); rabbit-anti-Uromodulin(THP) antibody (1:50, Proteintech, 11911-1-AP); Anti-UMOD antibody (1:50, Abcam, Ab207170); Calbindin-D28k Polyclonal antibody (1:100, Proteintech, 14479-1-AP); mouse-anti-CK19 antibody (1:50, Abcam, ab220193); rabbit-anti-CK19 antibody (1:500, Abcam, Ab133496). goat anti-Mouse IgG (H+L) Cross-Adsorbed Secondary Antibody, Alexa Fluor™ 594 (1:500, Invitrogen, A11005); goat anti-Rabbit IgG (H+L) Cross-Adsorbed Secondary Antibody, Alexa Fluor™ 488 (1:500, Invitrogen, A11008); donkey anti-Rabbit IgG (H+L) Highly Cross-Adsorbed Secondary Antibody, Alexa Fluor™ Plus 594 (1:500, Invitrogen, A32754); donkey anti-Rabbit IgG (H+L) Highly Cross-Adsorbed Secondary Antibody, Alexa Fluor™ 488 (1:500, Invitrogen, A21206).

#### Western blot

Rat liver tissues were homogenized in RIPA buffer (50 mM Tris pH 7.4, 150 mM NaCl, 1% deoxycholate, 0.1% SDS, 1% Triton-X-100) supplemented with protease and phosphatase inhibitors (Beyotime, P1050) on ice for 30 minutes. Lysates were centrifuged at 12,000 g for 15 minutes at 4°C, and the supernatants were mixed with 2X SDS-loading buffer and boiled at 100°C for 10 minutes. Proteins were separated on 10% SDS-PAGE gels and transferred to Immobilon-P PVDF Membranes (Millipore, IPVH00010). The membranes were blocked with 5% non-fat Powdered Milk (BBI, A600669-0250) for 1 hour at room temperature, incubated with primary antibody diluted in blocking buffer overnight at 4°C, and then with HRP-conjugated secondary antibodies for 1 hour at room temperature.

The flowing antibodies were used: rabbit-anti-alpha-SMA antibody (1:2000, Affinity, AF1032); rabbit-anti- PDGFR beta antibody (1:200, Proteintech, 13449-1-AP); rabbit-anti-Cdk1 antibody (1:2000, Proteintech, 19532-1-AP); rabbit-anti-Ccna2 antibody (1:5000, Proteintech, 18202-1-AP); rabbit-anti-Cleaved Casepase3 antibody (1:2000, BD Bioscience, 559565); mouse-anti-PCNA antibody (1:2000, Proteintech, 60097-1-1g); rabbit-anti-Hsp90 antibody (1:2000, Proteintech, 13171-1-AP); goat anti-rabbit IgG H&L/ HRP (1:5000, Bioss, bs-0295G-HRP).

#### Validation anti-Cdk1 and anti-Ccna2 antibodies

*Cdk1* and *Ccna2* coding sequences were amplified from rat liver cDNA. An HA tag sequence was fused to the N-terminal of *Cdk1* gene, and GFP tag sequence was fused to the C-terminal of *Ccna2* gene. The tagged *Cdk1-HA* and *Ccna2-GFP* constructs were cloned into *pcDNA3.1(+)* vector at BamHI and HindIII restriction sites. *pcDNA3.1(+)*-*Cdk1*-*HA* and *pcDNA3.1* (+)-*Ccna2*-*GFP* constructs were transfected into HEK293T cells, and whole cell lysates were collected 24 hours post transfection for antibody validation.

#### RNA sequence

Livers from three male WT and three male *Pkhd1*^*-/-*^ rats were collected for RNA sequencing. Total RNA was extracted using TRIzol reagent (Invitrogen) according to the manufacturer’s instructions. Briefly, samples were homogenized in 1 ml TRIzol and centrifuged to remove debris. Phase separation was induced with 0.2 ml chloroform followed by centrifugation at 12,000 rpm for 15 min at 4°C. RNA was precipitated from the aqueous phase using isopropanol, washed with 75% ethanol, and dissolved in RNase-free water. Paired-end sequencing was performed on an Illumina sequencing platform (Personalbio). Differential Gene Expression (DEG) analysis was conducted using DESeq with thresholds of |log2FoldChange| > 1 and P<0.05. Gene Ontology (GO) enrichment analysis was performed using topGO and Kyoto Encyclopedia of Genes and Genomes (KEGG) pathway enrichment analysis was using clusterProfiler. Hierarchical cluster and heatmap visualization were generated using the pheatmap package in R v4.1.2.

### Quantification and Statistical analysis

Statistical analyses were performed using GraphPad Prism 10. The Mann-Whitney U test was used for division angle comparison between *Pkhd1*^*CreER/+*^*;ZsGreen* and *Pkhd1*^*CreER/CreER*^*;ZsGreen* rats. Two-group comparison used unpaired Student’s t test, and multiple group comparisons were performed by two-way ANOVA followed by Tukey’s multiple comparison test. Data are presented as mean ± s.e.m., with significance defined as ∗∗∗*P* < 0.001; ∗∗*P* < 0.01; ∗*P* < 0.05. Transcriptome data were analyzed and graphed using R (v4.1.2). Specific sample sizes (n) and the methods used for the random selection of quantification regions are detailed in the corresponding 'method details'.

### Additional resources

There is no additional resource associated with this article.

## Supplemental information

Data S1. Supplementary figures and figure legends

Table S1. Differentially expressed genes (DEGs) in the liver of P45 rats by RNA-seq, related to Figure S11

Table S2. Primers for qPCR
