# Supplementary material for: Epithelial cell expansion drives cyst progression in genetic models of autosomal recessive polycystic kidney disease
Source: iScience. 2026 Jun 5;29(6):116288. doi: 10.1016/j.isci.2026.116288 (PMC13266189; doi:10.1016/j.isci.2026.116288)
Supplement: Document S1. Figures S1–S14 [file mmc1.pdf]

iScience, Volume *29*

Supplemental information

Epithelial cell expansion drives cyst progression in genetic models of autosomal recessive polycystic kidney disease

Shuncheng Liu, Xiaole Chen, Zhaoning Liu, Jun Tang, Haoran Tian, Ying Peng, Xiao Mao, Ruping Dai, Bin Zhao, Xugui Li, Li Li, Lingfei Luo, and Ming Ma

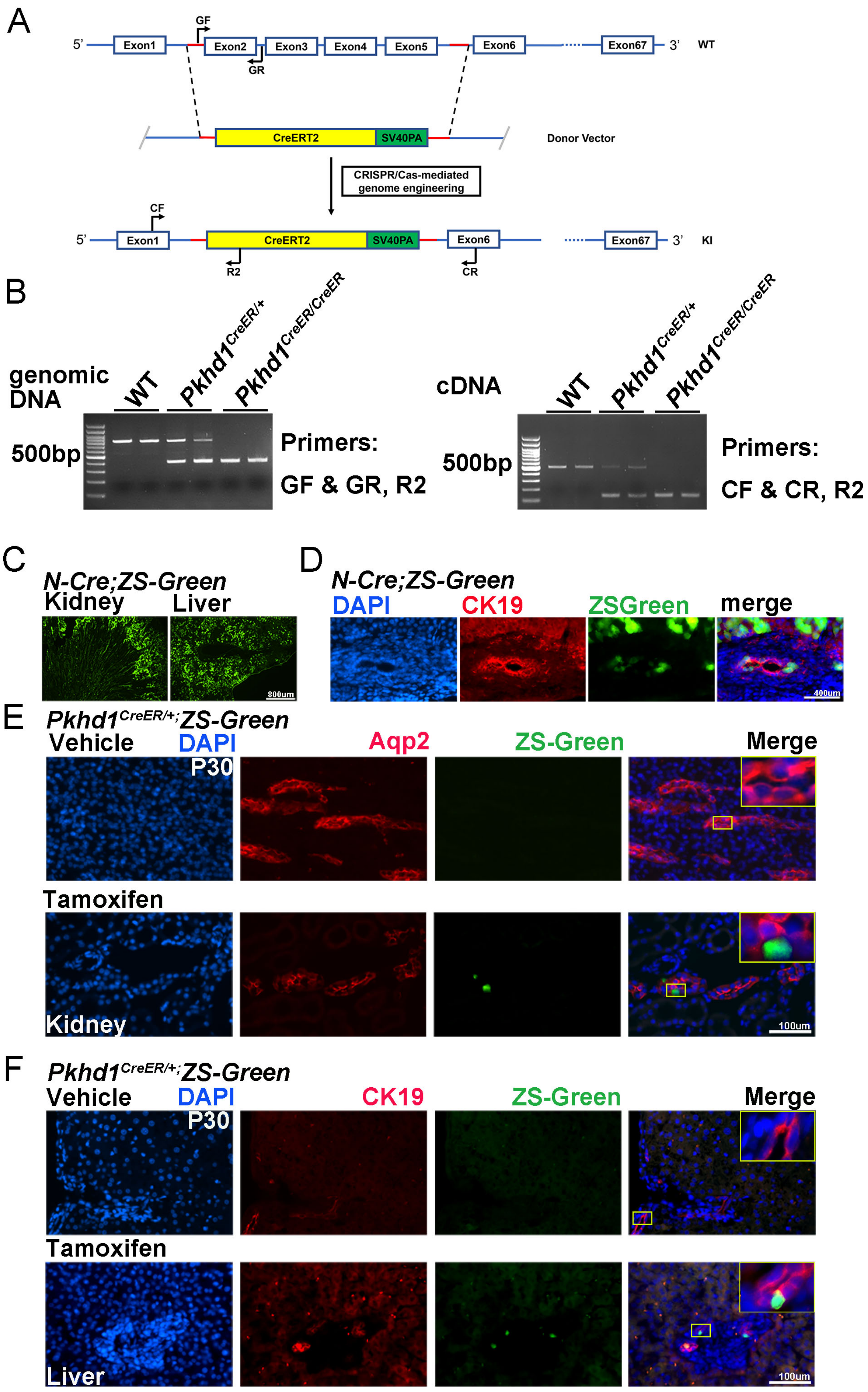

Figure S1. Validation of *Pkhd1^{CreER}* rat and reporter assay for *Pkhd1^{CreER}* rat.

A. Schematic drawing of WT and *Pkhd1^{CreER}* allele. CreER was inserted after ATG on exon2,

replacing exon 2-5 of *Pkhd1.* GF, GR, R2 are used as primers to amplify genomic DNA, CF, CR, R2 are used as primers to amplify cDNA. **B.** Gel electrophoresis of PCR amplicons to identify WT and *Pkhd1*^{CreER} alleles. Using combination of GF, GR and R2 primers, genomic DNA as template, WT rat generates an amplicon of 770 bp, *Pkhd1*^{CreER} allele generates an amplicon of 414 bp. Using combination of CF, CR and R2 primers and cDNA as template, WT rat generates an amplicon of 563 bp, which represent amplicon with primers CF and CR, *Pkhd1*^{CreER} allele generates an amplicon of 154 bp, which represent amplicon with primers CF and R2. **C**. Images of the kidney and liver sections of *CAG-Ncre;ZS-Green* rats. **D**. Immuno-fluorescence of anti-CK19 (red) antibody on liver sections of *CAG-Ncre;ZS-Green* rat. **E**. Immuno-fluorescence of anti-Aqp2 (red) on kidney sections of *Pkhd1*$^{CreER/+}$;*ZS-Green* rats at P30 induced with corn oil or tamoxifen with doses of 200mg/kg intraperitoneally injected at P5. **F**. Immuno-fluorescence of anti-CK19 (red) on liver sections of *Pkhd1*$^{CreER/+}$;*ZS-Green* rats at P30 induced with corn oil or tamoxifen intraperitoneally injected with doses of 200mg/kg into experimental rats at P5. Scale bars, 800 μm (**C**); 400 μm (**D**); 100 μm (**E, F**).

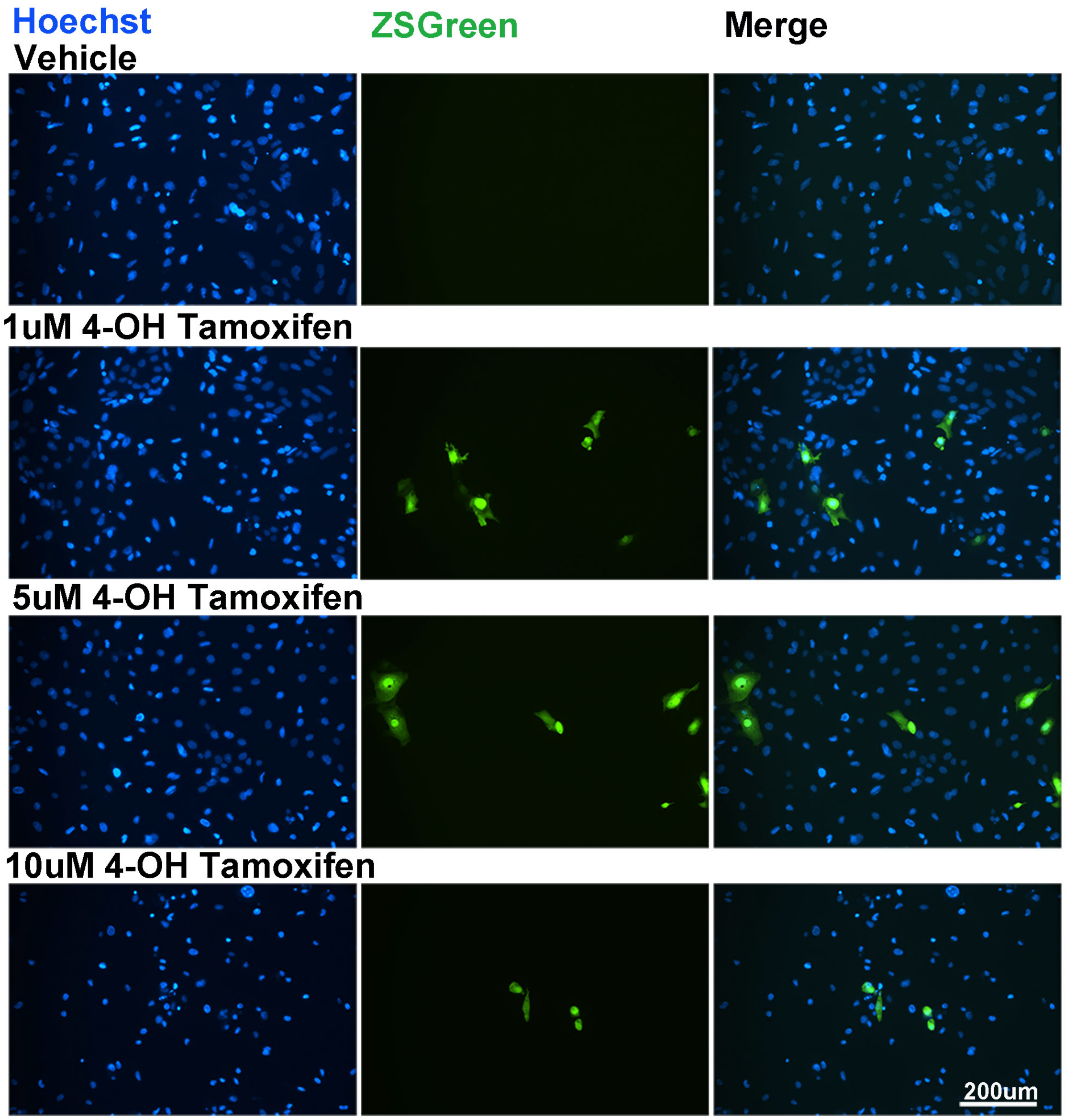

Figure S2. Primary kidney cells from *Pkhd1$^{CreER/+}$;ZsGreen* rats treated with 4OH-tamoxifen

Image of primary kidney cells from *Pkhd1$^{CreER/+}$;ZsGreen* rats treated with vehicle or 1, 5, 10 μM 4OH-tamoxifen for 16 hours. Cells were counterstained with DAPI or Hoechst to visualize nuclei. Scale bars, 200 μm.

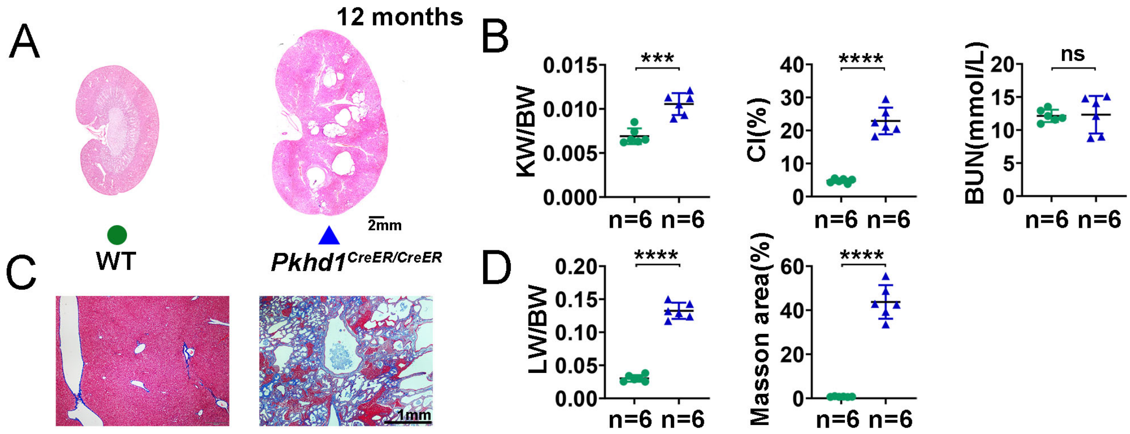

Figure S3. Polycystic kidney and liver disease development in *Pkhd1*$^{CreER/CreER}$ rats.
A–B. Scanned images of H&E stained kidney sections (**A**) and aggregated data of the kidney body weight ratio (**B**), cystic index and blood urea nitrogen (BUN) of 1-year-old WT (n = 6), and *Pkhd1*$^{CreER/CreER}$ (n = 6) rats. **C–D**. Masson trichrome stained liver sections of the 1-year-old rat of WT and *Pkhd1*$^{CreER/CreER}$ rats (**C**) and aggregated data of the liver body weight ratio, percentage of Masson area to parenchyma (**D**) of WT (n = 6) and *Pkhd1*$^{CreER/CreER}$ (n = 6) rats. Comparisons were performed by T-test, presented as mean ± s.e.m. *P < 0.05; **P < 0.01; ***P < 0.001; Scale bars, 2 mm (**A**); 1 mm (**C**).

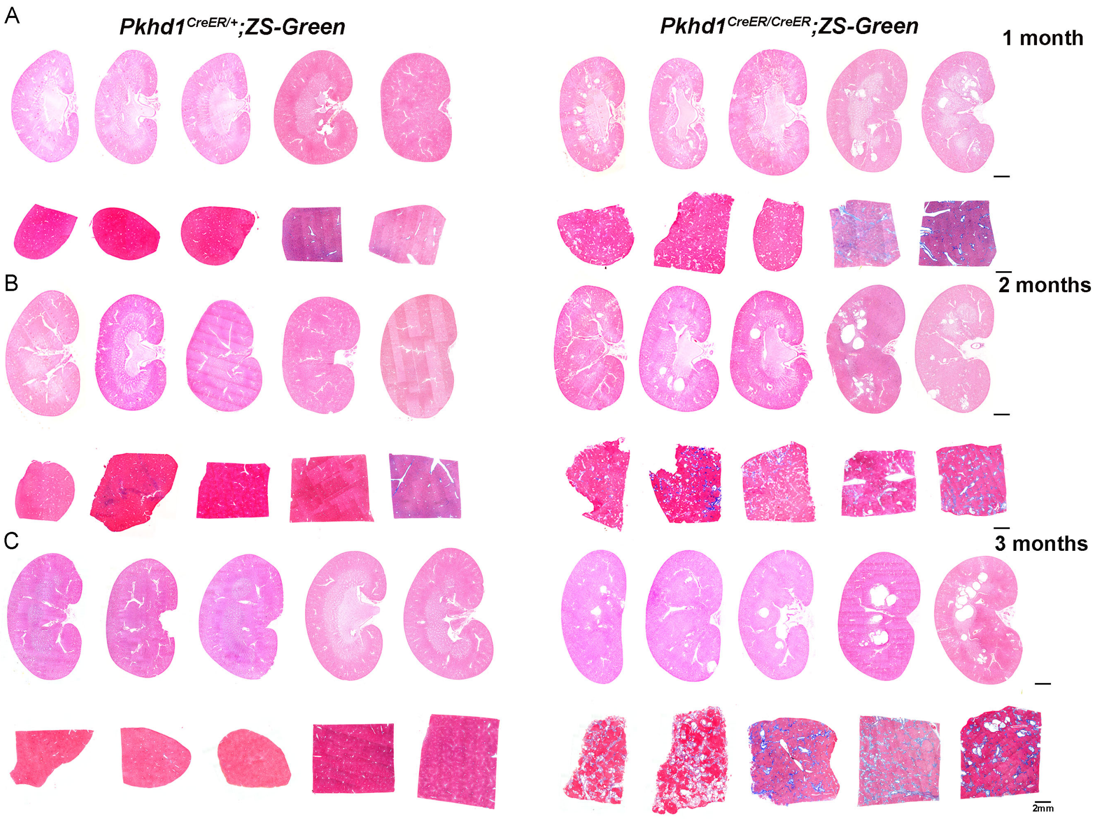

Figure S4. Collage of kidney and liver sections of *Pkhd1*$^{CreER/+}$ and *Pkhd1*$^{CreER/CreER}$ rat at P30, P60, and P90.

Scale bars, 2 mm.

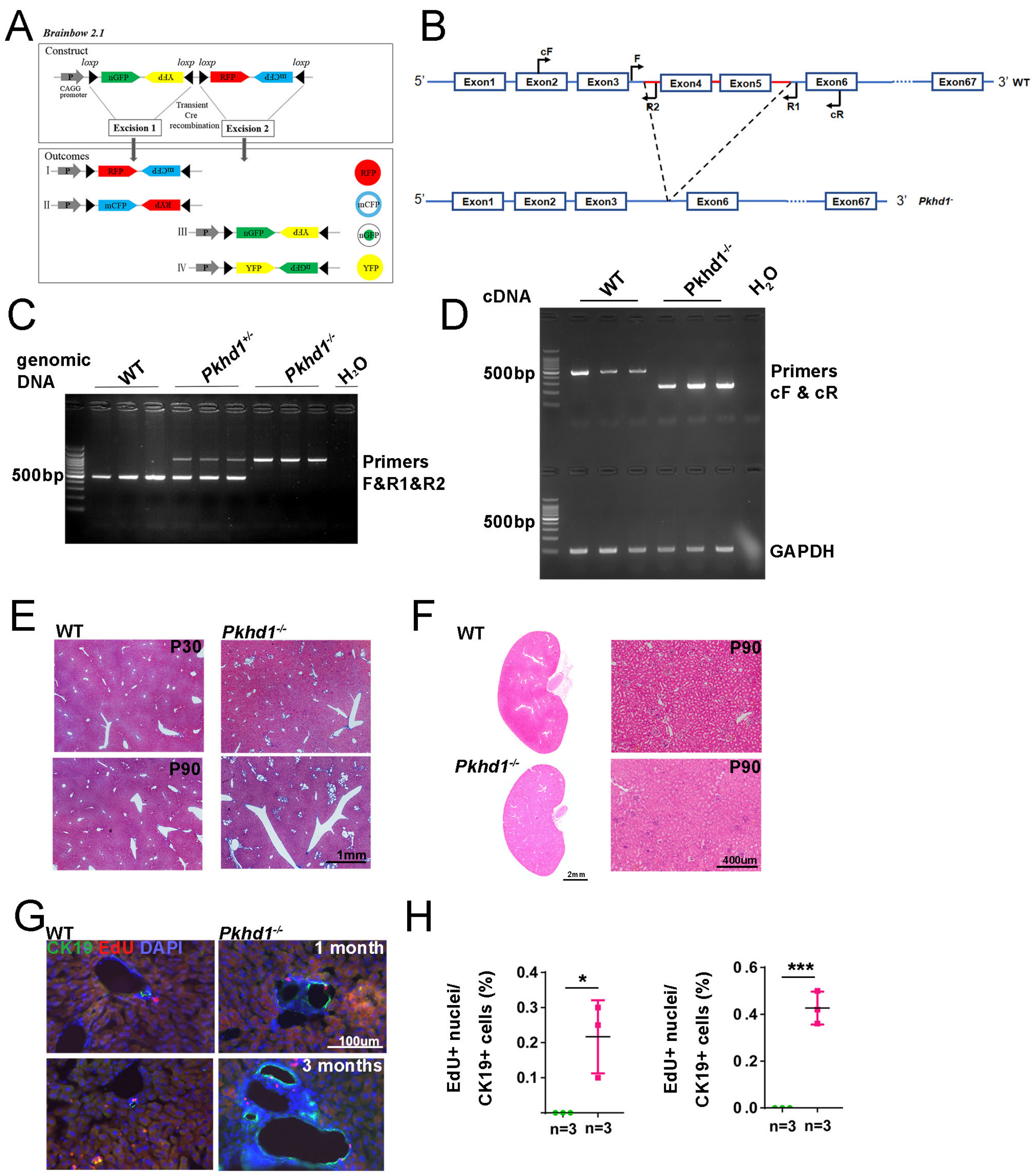

Figure S5. Combine *Rosa^{brainbow}*, *CK19^{CreER}*, and *Pkhd1$^{-/-}$* mice to do lineage tracing of cholangiocytes.

A. Schematic drawing of *Rosa$^{Brainbow2.1}$* allele. Cre-mediated recombination generates RFP, mCFP, nGFP and YFP, 4 fluorophores in a stochastic manner. **B**. Schematic drawing of WT and *Pkhd1^{-}* alleles. Intron 3 to intron 5 were knock out region. Forward primer F and reverse primer R2 on intron 3, R1 is the reverse primer on intron 5. CRISPR/Cas9 mediated genome editing generates *Pkhd1^{-}* allele. **C.** Gel electrophoresis of PCR amplicon to genotype WT and *Pkhd1^{-}* alleles. Use combination of F, R1 and R2 primers, genomic DNA as template, WT mice generate an amplicon of 487 bp, null mice generate an amplicon of 939 bp. **D**. Using cF, cR primers and cDNA as template, WT mice generated an amplicon of 507 bp, null mice generated an amplicon of 256 bp. **E**. Scanned image of Masson trichrome-stained WT and *Pkhd1$^{-/-}$* liver sections at 1 month and 3 months. **F**. Scanned image of

hematoxylin-eosin-stained WT and *Pkhd1*$^{-/-}$ kidney sections at 3 months. **G**. Immuno-fluorescence using anti-EdU and anti-CK19 antibodies on liver sections of WT and *Pkhd1*$^{-/-}$ mice at 1 month and 3 months injected with EdU 3 hours before sacrifice. **H**. Aggregated data of the ratio of EdU+ nuclei in CK19+ of WT (n = 3) and *Pkhd1*$^{-/-}$ (n = 3) mice at 1 month and 3 months. Comparisons were performed by *T*-test, presented as mean ± s.e.m. *$P < 0.05$; **$P < 0.01$; ***$P < 0.001$; Scale bars, 1 mm (**E**); 2 mm (**F left panel**); 400 µm (**F right panel**); 100 µm (**G**).

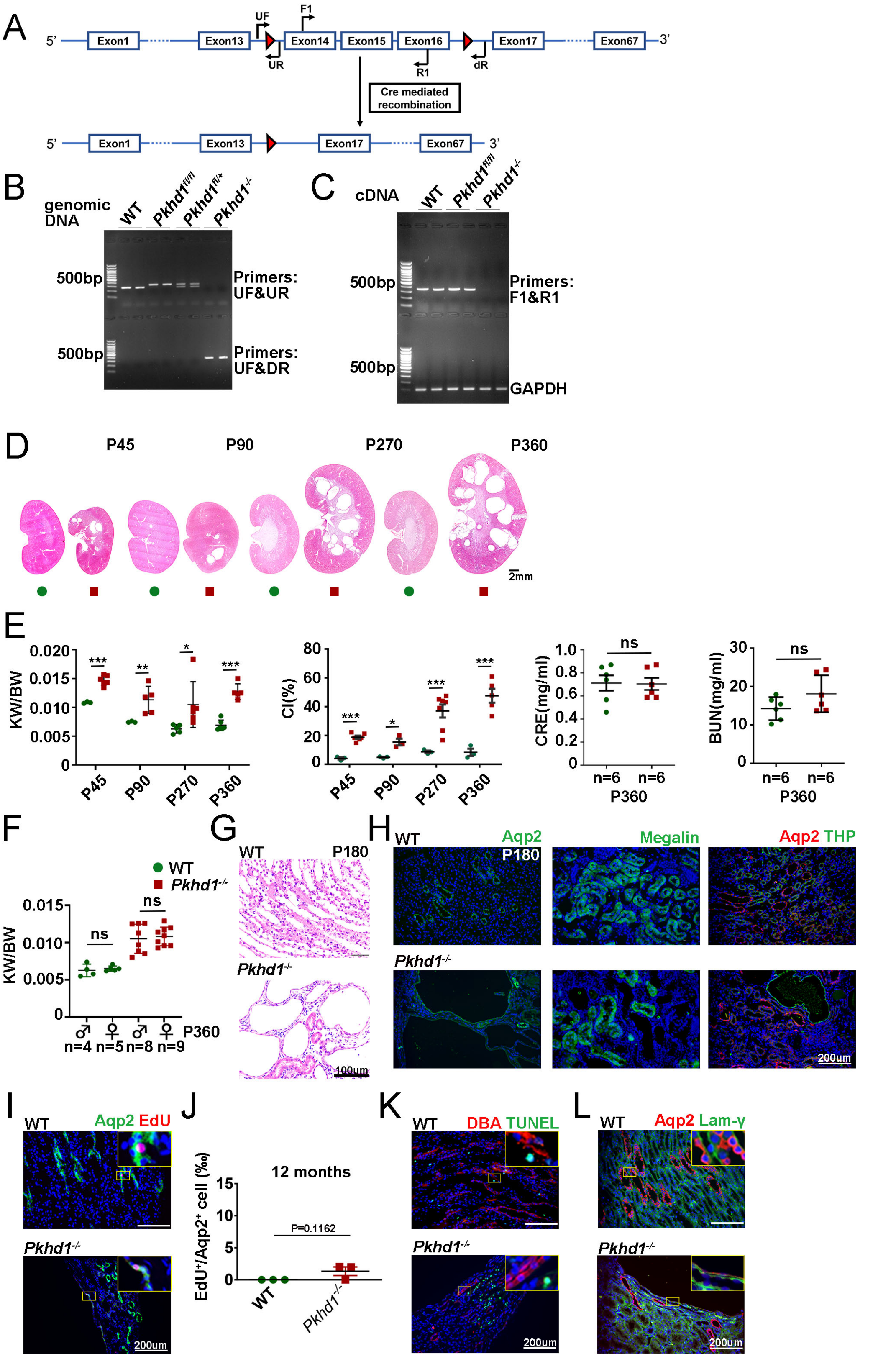

Figure S6. Generation of *Pkhd1*^{fl} and *Pkhd1*$^{-}$ alleles and characterize renal phenotype of *Pkhd1*$^{-/-}$ rat

A. Schematic drawing of WT, *Pkhd1*^{fl} and *Pkhd1*$^{-}$ alleles. Intron 13 and intron 16 were selected

as *flox* insertion sites. Forward primer uF and reverse primer uR flank the 5' flox site on intron 13, dR is the reverse primer of the 3' flox site on intron 16. F1 is the forward primer on exon 14, R1 is the reverse primer on exon 16. CRISPR/Cas9 mediated genome editing generates *Pkhd1*^{fl} allele, *Pkhd1*^{fl} allele crossed with Cre recombinase expression line to generate *Pkhd1*$^{-}$ allele. **B.** Gel electrophoresis of PCR amplicon to genotype WT, *Pkhd1*^{fl} and *Pkhd1*$^{-}$ alleles. Using combination of uF and uR primers, genomic DNA as template, WT rat generates an amplicon of 271 bp, *Pkhd1*^{fl} allele generates an amplicon of 333 bp, null rat generates no PCR product. **C.** Use combination of F1 and R1 primers, and cDNA as template, WT and *Pkhd1*$^{fl/fl}$ rats generate an amplicon of 350 bp, *Pkhd1*$^{-/-}$ rats generate no amplicon, while GAPDH generates an amplicon of 107 bp in WT, *Pkhd1*$^{fl/fl}$ and *Pkhd1*$^{-/-}$ rats. **D.** Scanned images of hematoxylin-eosin-stained kidney sections of WT and *Pkhd1*$^{-/-}$ rats at P45, P90, P270 and P365. **E.** Aggregated data of kidney body weight ratio and cystic index of WT (n = 3) and *Pkhd1*$^{-/-}$ (n = 6) rats at P45, WT (n = 3) and *Pkhd1*$^{-/-}$ (n = 5) rats at P90, WT (n = 5) and *Pkhd1*$^{-/-}$ (n = 6) rats at P270, and WT (n = 6) and *Pkhd1*$^{-/-}$ (n = 5) rats at P365, and aggregated data of serum urea nitrogen and serum creatinine of WT (n = 6) and *Pkhd1*$^{-/-}$ (n = 6) rats at P365. **F**. Comparison of kidney body weight ratio of male and female rats of WT (male: n = 4, female n = 5) and *Pkhd1*$^{-/-}$ (male: n = 8, female: n = 9) rats at P360 reveal that there is no gender dimorphism for cyst growth. **G.** HE stained kidney sections of WT and *Pkhd1*$^{-/-}$ rats at P180. **H.** Immuno-fluorescence of anti-Aqp2 (green), and anti-Megalin antibody (green) staining of kidney sections of WT and *Pkhd1*$^{-/-}$ rats at P180, the sections were counterstained with DAPI. Immuno-fluorescence of anti-Aqp2 (red) and anti-THP (green) antibody staining of kidney sections of WT and *Pkhd1*$^{-/-}$ rats at P180, the sections were counterstained with DAPI. **I**. Immuno-fluorescence of anti-Aqp2 (green) and anti-EdU (red) antibody on kidney sections of WT and *Pkhd1*$^{-/-}$ rats injected with EdU 3 hours before sacrifice. **J**. Aggregated data of the ratio of EdU+ in Aqp2+ in *Pkhd1*$^{-/-}$ (n = 3) and WT (n = 3) rats at 12 months. **K**. TUNEL assay (green) and DBA (red) staining on kidney sections of WT and *Pkhd1*$^{-/-}$ rats. **L**. Immuno-fluorescence of anti-Laminin-γ1 (green) and anti-Aqp2 (red) antibody on kidney sections of WT and *Pkhd1*$^{-/-}$ rats. Comparisons were performed by *T*-test, presented as mean ± s.e.m. *P < 0.05; **P < 0.01; ***P < 0.001; Scale bars, 2 mm (**D**); 100 μm (**G**); 200 μm (**H, I, K, L**).

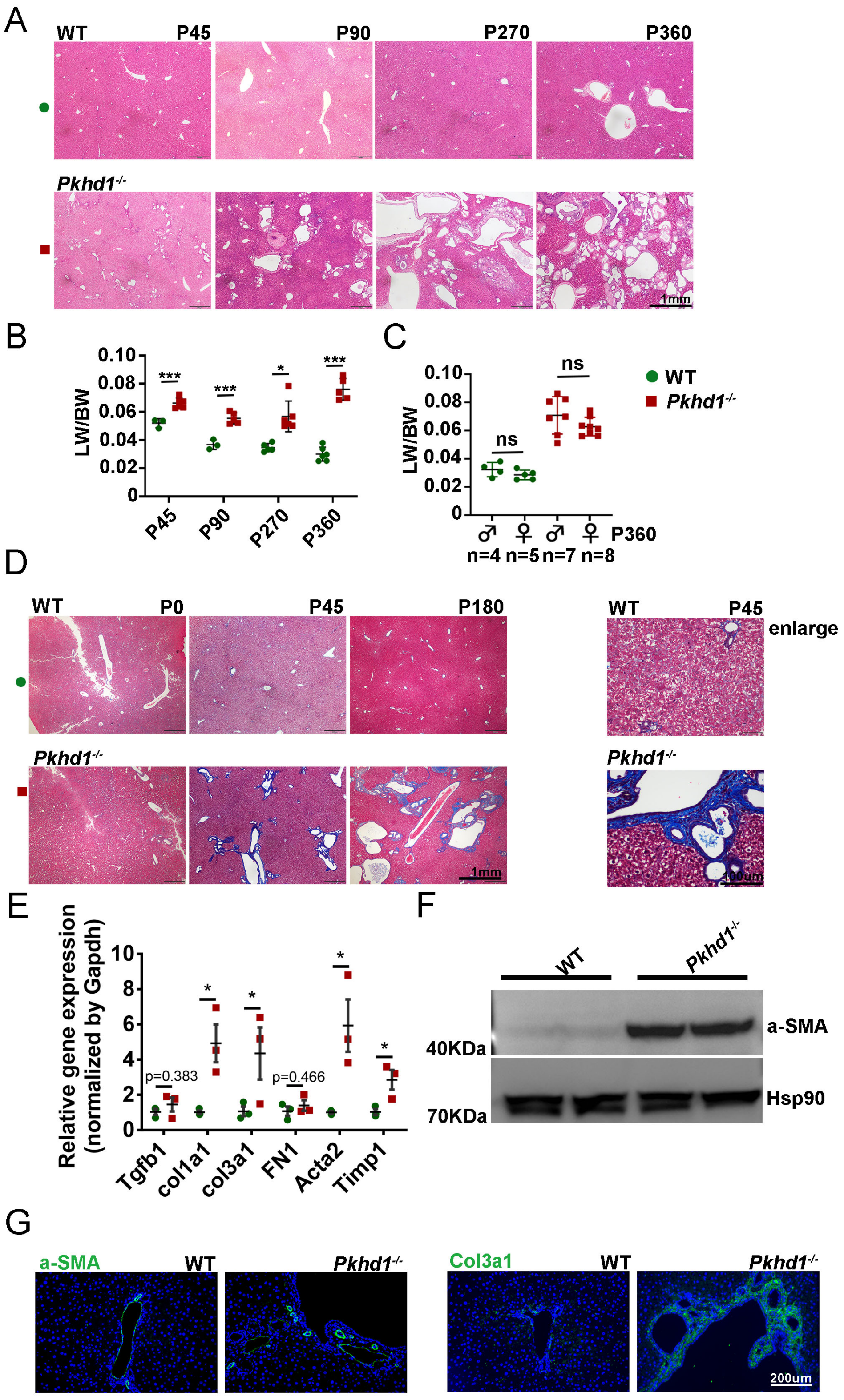

Figure S7. Fibrocystic liver disease in *Pkhd1*$^{-/-}$ rat

A. HE staining of liver sections of WT and *Pkhd1*$^{-/-}$ rats at P45, P90, P270, P365. **B.** Aggregated data of liver body weight ratio of WT (n = 3) and *Pkhd1*$^{-/-}$ (n = 6) rats at P45, WT (n = 3) and *Pkhd1*$^{-/-}$ (n = 5) rats P90, WT (n = 5) and *Pkhd1*$^{-/-}$ (n = 6) rats P270, WT (n = 6) and *Pkhd1*$^{-/-}$ (n = 5) rats P365. **C.** Comparison of liver body weight ratio of male and female rats of WT (male: n = 4, female: n = 5) and *Pkhd1*$^{-/-}$ rats (male: n = 7, female: n = 8) at P360 revealed

that there is no gender dimorphism for cyst growth. **D**. Masson trichrome staining of liver sections of WT and *Pkhd1*$^{-/-}$ rats at P0, P45, P180. Enlarged images on the right panel at P45. **E**. Validation of fibrosis-related genes of WT (n = 3) and *Pkhd1*$^{-/-}$ (n = 3) rats' liver by qPCR at P45. **F**. Western blot using anti-a-SMA and anti-Hsp90 antibodies on liver tissues of WT and *Pkhd1*$^{-/-}$ rats at P45. **G**. Immuno-fluorescence of anti-a-SMA or anti-col3a1 antibodies staining on liver sections of WT and *Pkhd1*$^{-/-}$ rats at P45. Comparisons were performed by *T*-test, presented as mean ± s.e.m. *P < 0.05; **P < 0.01; ***P < 0.001; Scale bar, 1 mm (**A** and **D**); 100 μm (enlarge **D**); 200 μm (**G**).

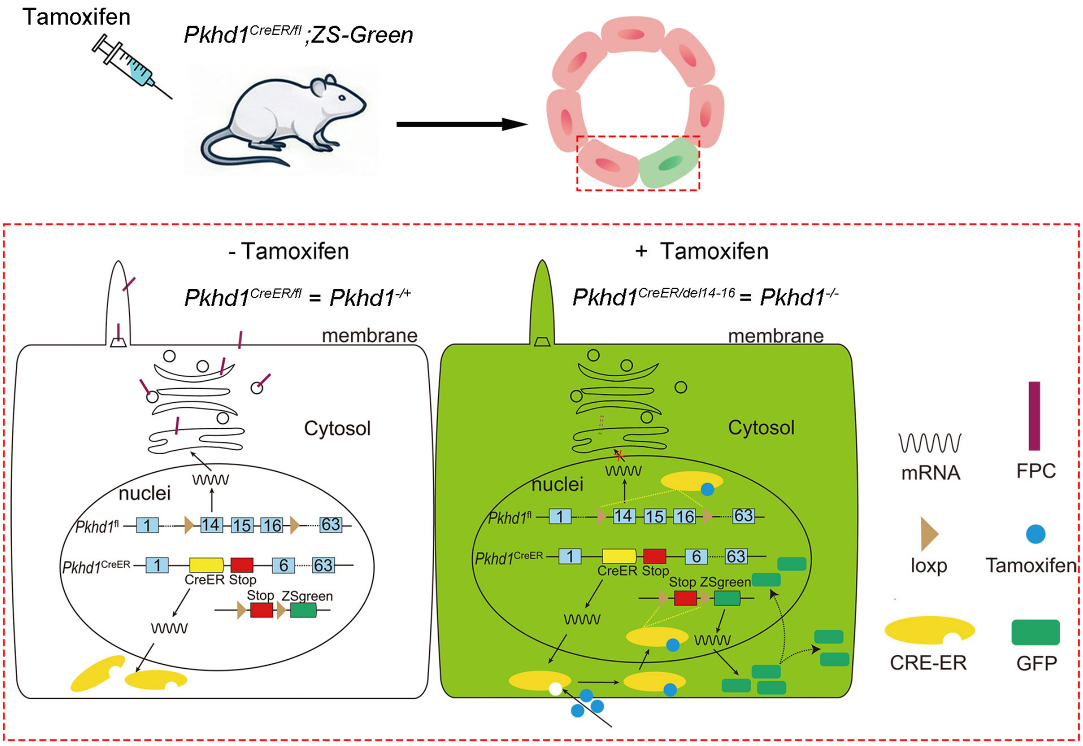

Figure S8. Schematic diagram of the mosaic analysis in *Pkhd1*$^{CreER/fl}$*;ZS-Green* rats
Mosaic analysis was conducted using *Pkhd1*$^{CreER/fl}$*;ZsGreen* rats. The *Pkhd1*^{CreER} allele functions as a null allele, whereas the *Pkhd1*^{fl} allele contains *loxP* sites flanking exons 14-16 and functions as a wild-type allele before recombination. To induce mosaicism, pups received intraperitoneal injections of tamoxifen (200 mg/kg) at P5-P6. In cells with effective Cre activity, the excision of exons 14–16 leads to a frameshift and gene inactivation, converting the *Pkhd1*^{fl} allele into a null allele (*Pkhd1*$^{del14-16}$) and activating the ZsGreen reporter. This results in ZsGreen-positive cells with a homozygous null genotype (*Pkhd1*$^{CreER/del14-16}$ equivalent *Pkhd1*$^{-/-}$), while adjacent ZsGreen-negative cells remain heterozygous (*Pkhd1*$^{CreER/fl}$ equivalent *Pkhd1*$^{-/+}$). This strategy allows for the distinction of *Pkhd1*-deficient and *Pkhd1*-heterozygous cells within the same tubule.

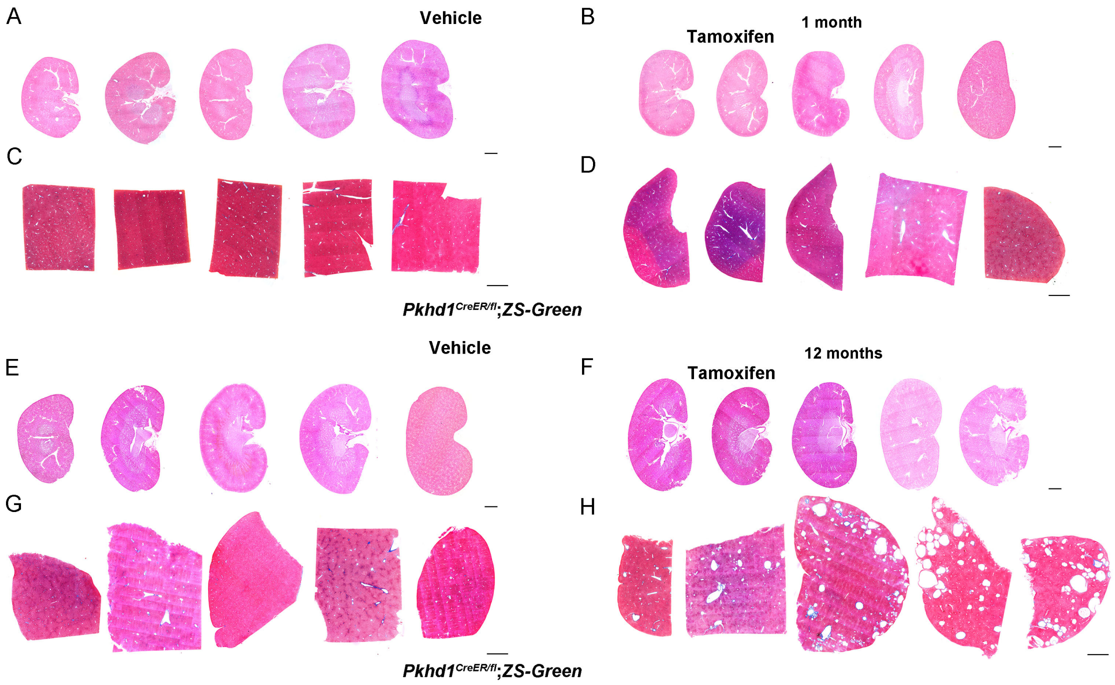

Figure S9. Collage of scanned images of 1-month-old and 1-year-old *Pkhd1*$^{CreER/fl}$ rat kidney and liver sections induced with corn oil or tamoxifen at P5 and P6.
Scale bars: 2 mm (**A, B, E, F**); 5 mm (**C, D, G, H**).

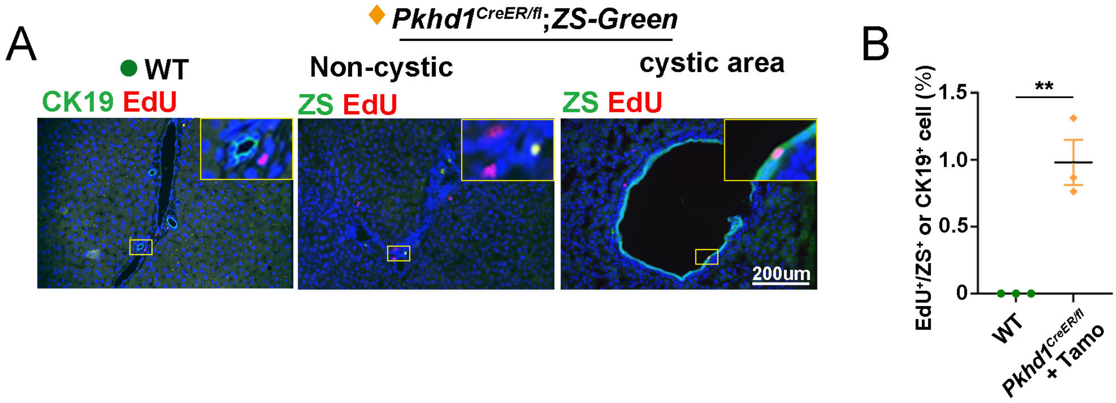

Figure S10. Proliferation and apoptosis assay for cyst formation in *Pkhd1$^{CreER/fl}$* rats and *Pkhd1$^{CreER/CreER}$* rats.

A. Immuno-fluorescence using anti-EdU antibody on liver section of WT and *Pkhd1$^{CreER/fl}$;ZS-Green* rat injected with EdU 3 hours before sacrifice. **B**. Aggregated data of the ratio of EdU+ within CK19+ or the ratio of EdU+ in ZSGreen-positive nuclei of the livers of WT (*n* = 3) and *Pkhd1$^{CreER/fl}$;ZS-Green* (*n* = 3). Comparisons were performed by *T*-test, presented as mean ± s.e.m. *$P < 0.05$; **$P < 0.01$; ***$P < 0.001$; Scale bars, 200 μm (**A**).

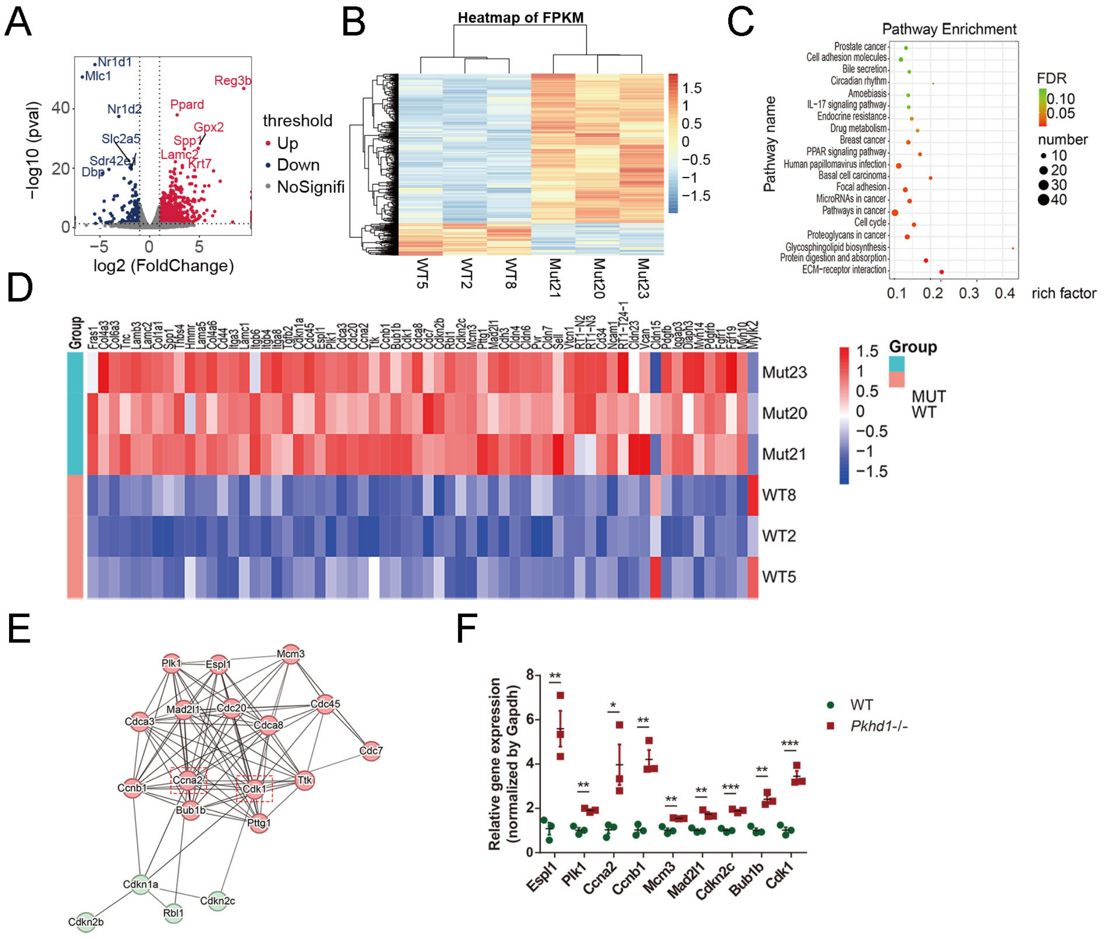

Figure S11. Transcriptomics of early fibrocystic liver in *Pkhd1*$^{-/-}$ rats.

A. Volcano plots of Differential expressed genes (DEGs) detected between *Pkhd1*$^{-/-}$ (n = 3) and WT (n = 3) rat liver at P45. Threshold = $P \leqslant 0.05$ & |Foldchange| $\geqslant 2$. **B**. Heatmap of all DEGs expression levels in each rat liver. **C**. Top 20 of KEGG pathways by analyzing liver DEGs. **D**. Heatmap of the liver DEGs in cell cycle, ECM-receptor interaction, cell adhesion molecules, regulation of actin cytoskeleton pathways. **E**. Protein-Protein Interaction Networks (PPI) of liver DEGs in cell cycle. **F**. Validation of the expression of selected genes identified from DEGs by qPCR in WT (n = 3) and *Pkhd1*$^{-/-}$ (n = 3) livers. Two group comparisons were performed by T-test, presented as mean ± s.e.m. ***$P < 0.001$; **$P < 0.01$; *$P < 0.05$.

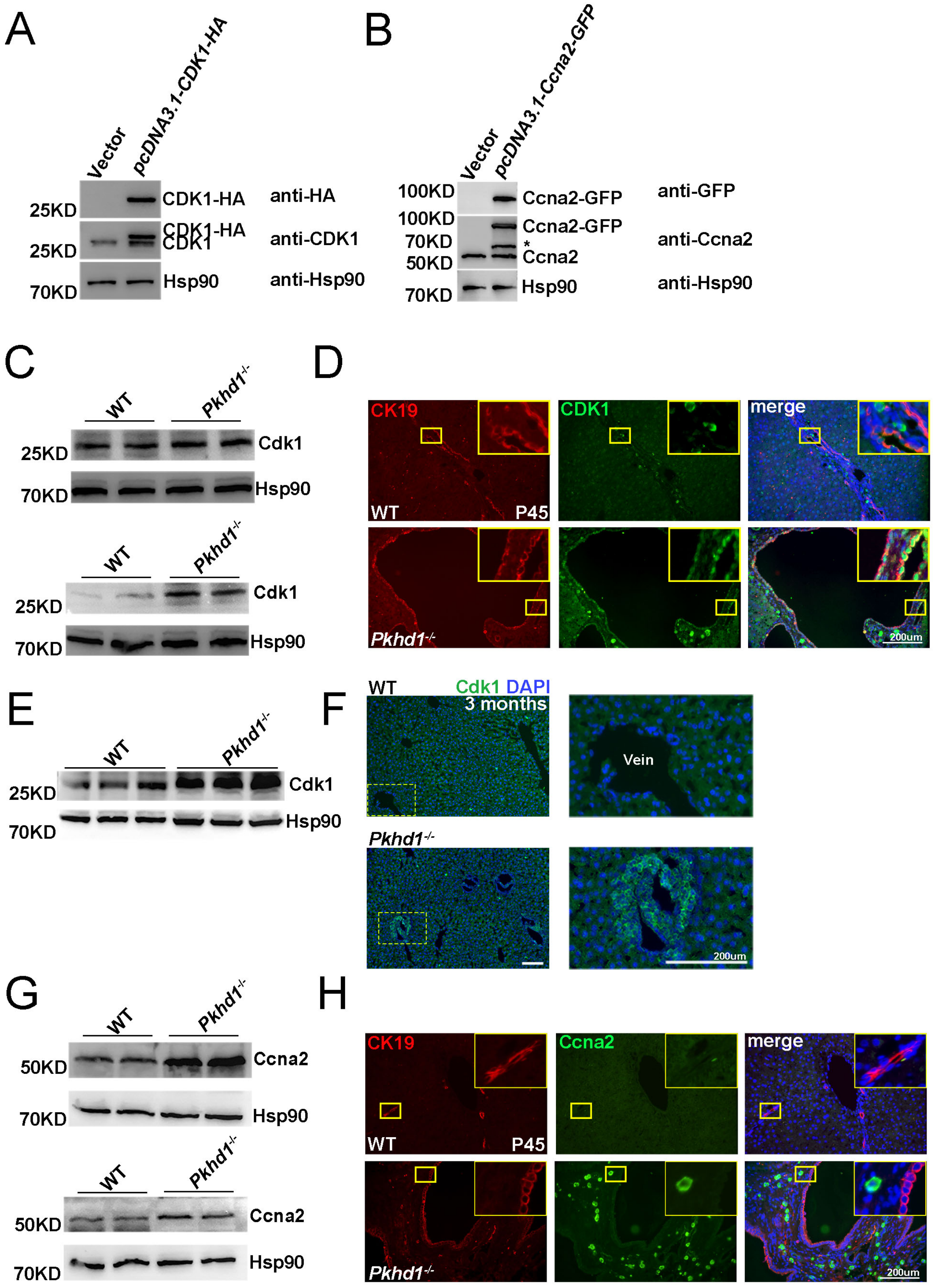

Figure S12. Expression of Cdk1 and Ccna2 in polycystic liver tissues.
A. Western blot analysis of HEK293T cells lysate transfected with *pcDNA3.1-Cdk1-HA* constructs or empty vector with anti-HA, anti-Cdk1, and anti-Hsp90 antibodies. Anti-HA antibody recognizes exogenously expressed Cdk1-HA fusion protein, and anti-Cdk1 antibodies recognize exogenously expressed Cdk1-HA fusion protein and endogenous Cdk1 protein. **B**. Western blot analysis the lysate of HEK293T cells transfected with *pcDNA3.1-Ccna2-GFP* constructs or empty vector with anti-GFP, anti-Ccna2, and anti-Hsp90 antibodies. Anti-GFP antibody recognizes exogenously expressed Ccna2-GFP fusion protein,

and anti-Ccna2 antibody recognizes exogenously expressed Ccna2-GFP fusion protein and endogenous Ccna2 protein. **C–D**. Analyzing the expression of Cdk1 protein in WT and *Pkhd1*$^{-/-}$ rat livers at P45 (upper panels) and P365 (lower panel) via western blot (**C**), and immuno-fluorescence (**D**) with anti-Cdk1 and anti-CK19 antibodies on the liver sections of WT and *Pkhd1*$^{-/-}$ rats at 45, sections were counterstained with DAPI. **E-F.** Analyzing the expression of Cdk1 protein in WT and *Pkhd1*$^{-/-}$ mouse livers at 3 months via western blot (**E**) and immuno-fluorescence (**F**) with anti-Cdk1 antibody on the liver sections of WT and *Pkhd1*$^{-/-}$ mouse at 3 months, sections were counterstained with DAPI. **G-H**. Analyzing the expression of Ccna2 protein in WT and *Pkhd1*$^{-/-}$ rat livers at P45 (upper panels) and P365 (lower panels) via western blot (**G**), and immuno-fluorescence (**H**) with anti-Ccna2 and anti-CK19 antibodies on the liver sections of WT and *Pkhd1*$^{-/-}$ rats at 45, sections were counterstained with DAPI. Scale bars, 200 µm (**D, F, and H**).

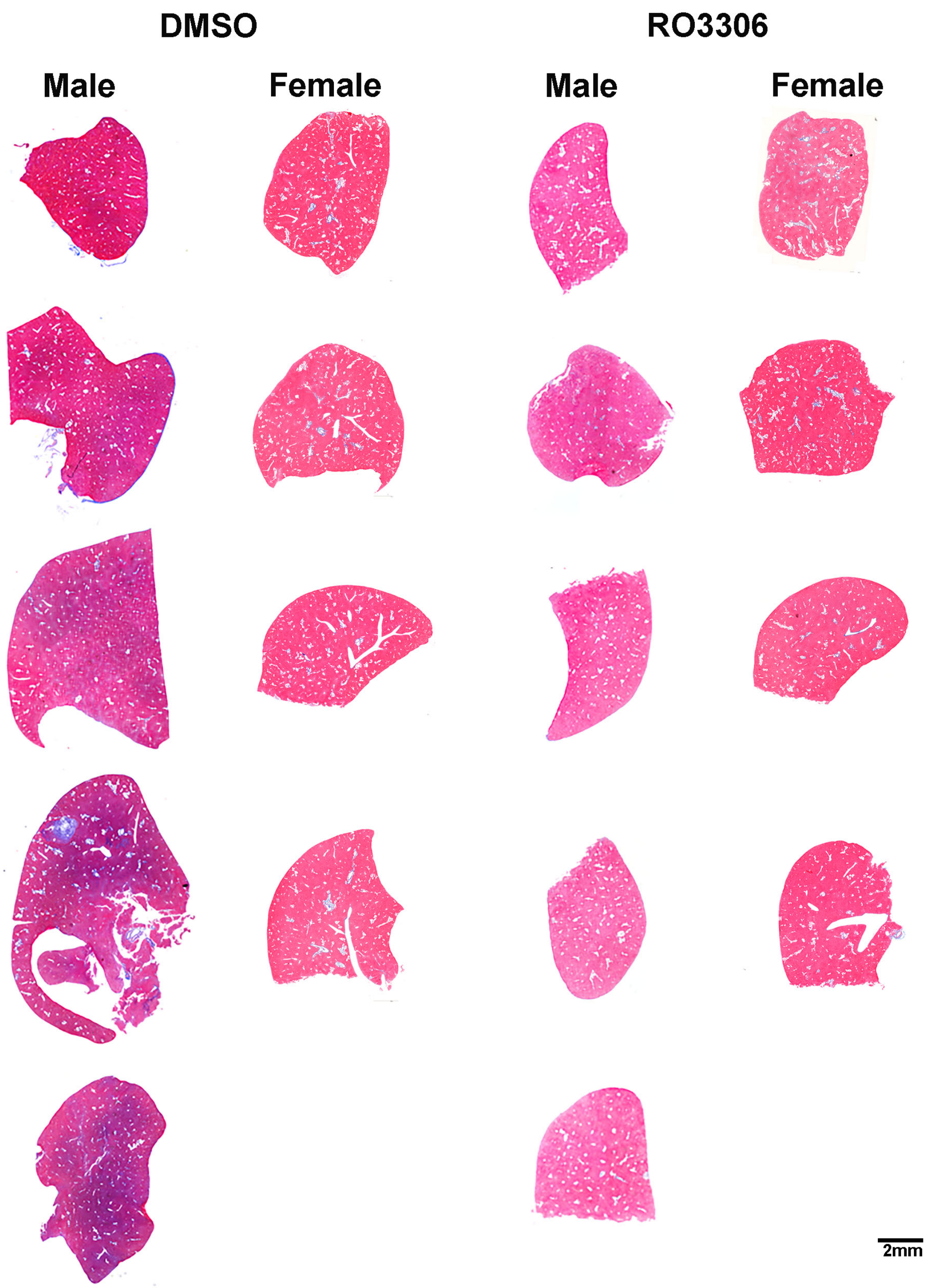

Figure S13. Collage of scanned images of *Pkhd1*$^{-/-}$ mice liver sections treated with DMSO or RO-3306 at 3 months

Scale bar, 2 mm.

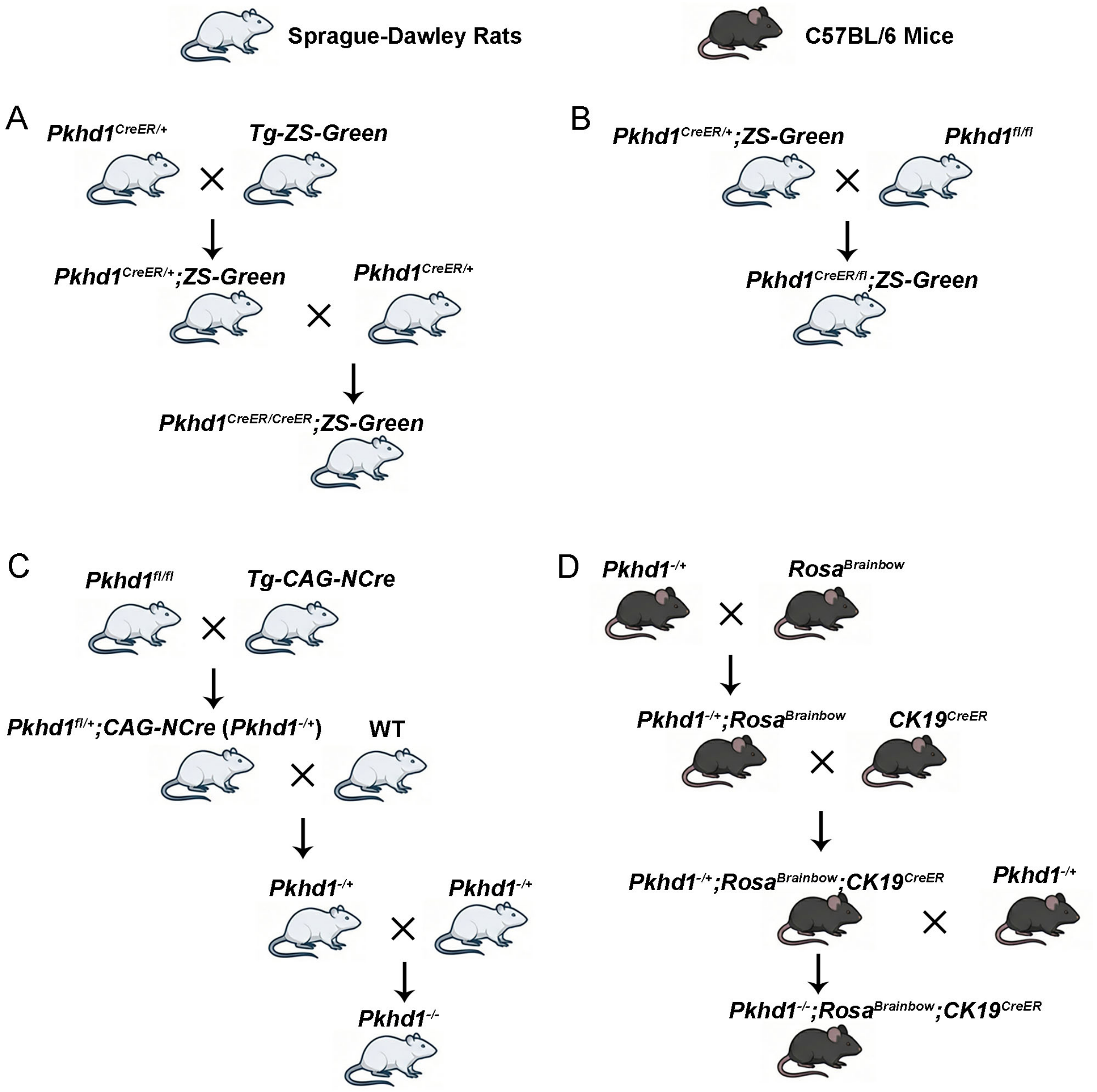

Figure S14. Schematic diagram of the breeding strategies for rats and mice
