# Supplementary material for: Epithelial cell expansion drives cyst progression in genetic models of autosomal recessive polycystic kidney disease
Source: iScience. 2026 Jun 5;29(6):116288. doi: 10.1016/j.isci.2026.116288 (PMC13266189; doi:10.1016/j.isci.2026.116288)

antibody validation

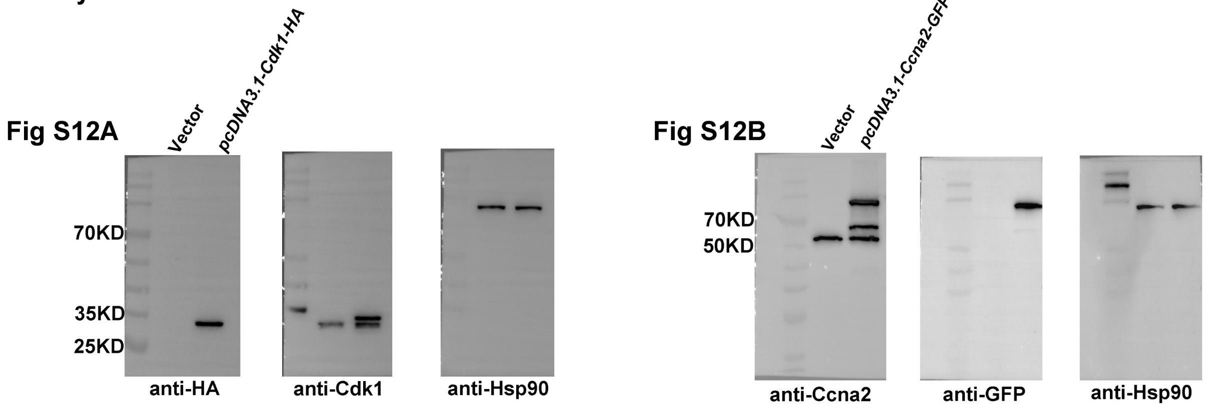

RO3306 Validation

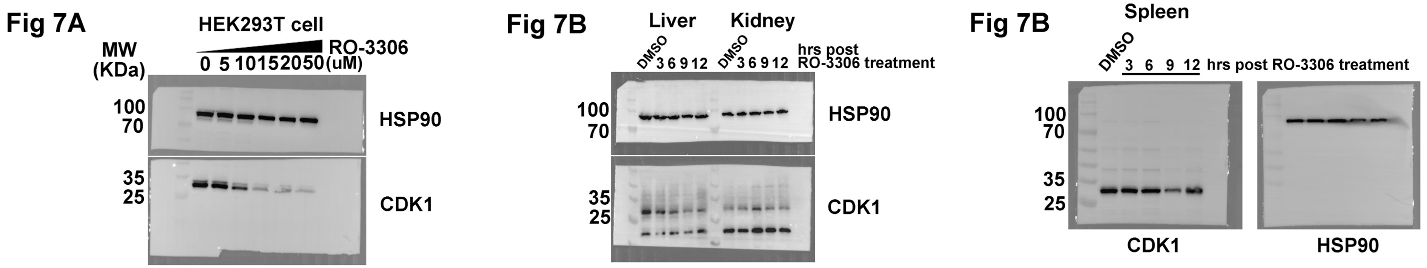

Rat liver

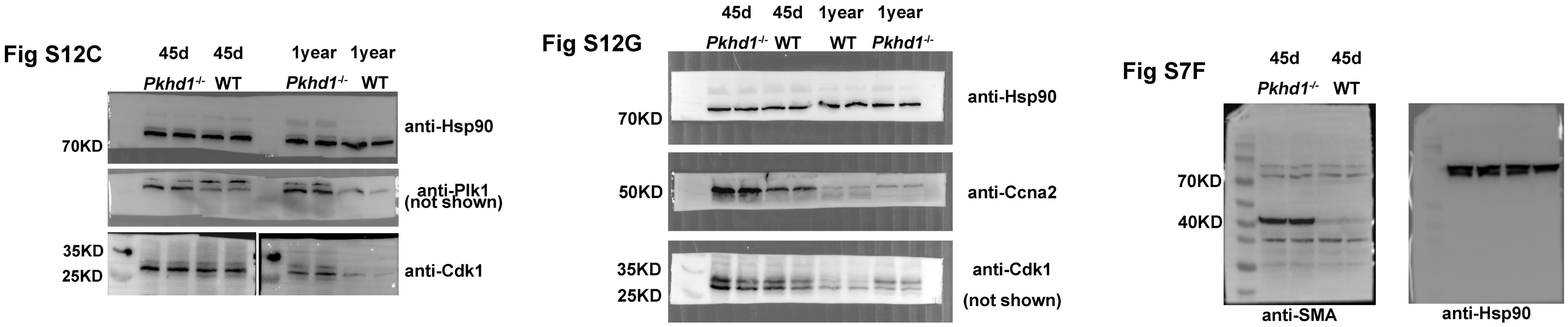

Mouse liver

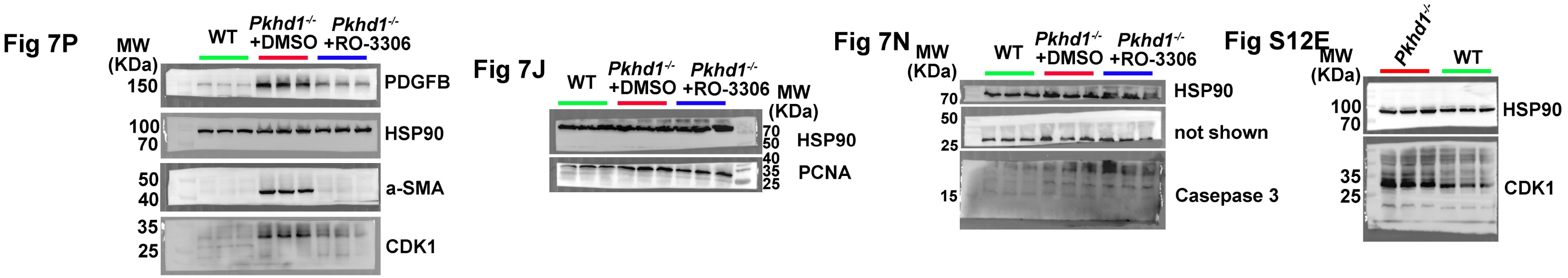

Supplement: Data S1. Supplementary figures and figure legends [file mmc2.pdf]
